# ChIP-Seq analysis identifies p27(Kip1)-target genes involved in cell adhesion and cell signalling in mouse embryonic fibroblasts

**DOI:** 10.1371/journal.pone.0187891

**Published:** 2017-11-20

**Authors:** Atilla Biçer, Serena Orlando, Abul B. M. M. K. Islam, Edurne Gallastegui, Arnaud Besson, Rosa Aligué, Oriol Bachs, Maria Jesús Pujol

**Affiliations:** 1 Department of Biomedical Sciences, University of Barcelona–IDIBAPS (Institut d’investigacions Biomèdiques August Pi i Sunyer), Barcelona, Spain; 2 Department of Genetic Engineering and Biotechnology, University of Dhaka, Dhaka, Bangladesh; 3 INSERM UMR1037, Cancer Research Center of Toulouse, Toulouse, France; 4 Université de Toulouse, Toulouse, France; 5 CNRS ERL5294, Toulouse, France; University of Minnesota Medical Center, UNITED STATES

## Abstract

The protein p27^Kip1^ (p27), a member of the Cip-Kip family of cyclin-dependent kinase inhibitors, is involved in tumorigenesis and a correlation between reduced levels of this protein in human tumours and a worse prognosis has been established. Recent reports revealed that p27 also behaves as a transcriptional regulator. Thus, it has been postulated that the development of tumours with low amounts of p27 could be propitiated by deregulation of transcriptional programs under the control of p27. However, these programs still remain mostly unknown. The aim of this study has been to define the transcriptional programs regulated by p27 by first identifying the p27-binding sites (p27-BSs) on the whole chromatin of quiescent mouse embryonic fibroblasts. The chromatin regions associated to p27 have been annotated to the most proximal genes and it has been considered that the expression of these genes could by regulated by p27. The identification of the chromatin p27-BSs has been performed by Chromatin Immunoprecipitation Sequencing (ChIP-seq). Results revealed that p27 associated with 1839 sites that were annotated to 1417 different genes being 852 of them protein coding genes. Interestingly, most of the p27-BSs were in distal intergenic regions and introns whereas, in contrast, its association with promoter regions was very low. Gene ontology analysis of the protein coding genes revealed a number of relevant transcriptional programs regulated by p27 as cell adhesion, intracellular signalling and neuron differentiation among others. We validated the interaction of p27 with different chromatin regions by ChIP followed by qPCR and demonstrated that the expressions of several genes belonging to these programs are actually regulated by p27. Finally, cell adhesion assays revealed that the adhesion of p27^-/-^ cells to the plates was much higher that controls, revealing a role of p27 in the regulation of a transcriptional program involved in cell adhesion.

## Introduction

Cell cycle progression is driven by different members of a family of serine/threonine kinases named cyclin dependent kinases (Cdks), characterized by their need for associating with a regulatory subunit, the cyclin, that potentiates Cdk catalytic activity [1]. Cdks involved in cell-cycle regulation include Cdk4, Cdk6 and Cdk2 that regulate progression through the G_1_ phase although additionally, Cdk2 also regulates S phase and Cdk1 that regulates mitosis [2] In early-mid G_1_ phase, cyclins-Cdk4/6 and cyclins-Cdk2 complexes phosphorylate and inactivate members of the retinoblastoma family of pocket proteins (pRb, p107 and p130) resulting in de-repression of multiple genes encoding for proteins required for DNA replication [3]. In addition to the regulation by cyclins, Cdk activity is also regulated by other mechanisms including phosphorylation, acetylation and binding to Cdk-inhibitors (CKIs)[1,4–6]. These mechanisms allow modulating, not only Cdk activity, but also its intracellular location and degradation.

Two families of CKIs have been described. The Cip/Kip family that includes p21^Cip1^ (p21), p27^Kip1^ (p27) and p57^Kip2^ that associate with most cyclin-cdk complexes involved in cell cycle regulation [7] and the INK4 family that includes p15^INK4B^, p16^INK4A^, p18^INK4C^ and p19 ^INK4D^ and that specifically acts on Cdk4 and Cdk6 [8].

All members of the Cip/Kip family of CKIs interact with both cyclin and Cdk subunits. Interestingly, it has been shown that the specific phosphorylation of tyrosine residues of p27 and p21 by members of the Src tyrosine kinase family, induces a conformational change that provokes a partial activation of the associated Cdk. Thus, under these circumstances cyclin-Cdk complexes might be partially active despite its association with p27 or p21 [9–13]. Hence, in fact these CKIs have to be considered modulators of Cdk activity better than only inhibitors.

During the last years it has been shown that p27 and p21 are involved in the regulation of transcription. This regulatory role is mediated by their association with specific transcription factors (TFs). Specifically, p27 directly interacts with p130 and E2F4 by its carboxyterminal moiety and acts as a transcriptional co-repressor of genes involved in cell cycle progression [14]. A recent paper describes a mechanistic model of how p27 regulates the expression of p130/E2F4 depending genes [15]. This model reveals that in early-mid G1 p27 recruits cyclin D2/D3-Cdk4/6 complexes on the promoters of target genes thus making its substrate p130 more affordable to Cdk complexes. The shift of Cdk from inactive to active can be achieved by Src mediated phosphorylation of specific tyrosines in p27 at mid G1. At that time after p130 phosphorylation, p27-cyclin D2/D3-Cdk4/6 complexes move out from the promoters and are substituted by p21-cyclin D1-Cdk2 that in a similar way is activated and additionally phosphorylates p130. As a consequence genes involved in cell cycle progression are de-repressed. This model highlights the collaboration between p27 and p21 in the transcriptional regulation of p130/E2F4-depending genes. In fact, a recent report indicates that p27 negatively regulates the expression of p21 during cell cycle, thus facilitating the time-dependent collaboration of both proteins in cell cycle regulation [16]. In addition to that, p27 may also regulate gene expression by association with other TFs as for instance Ets-1 [14]. Similarly, p21 behaves as a transcriptional regulator by associating with a number of TFs such as Myc, NRf2, CBP and STAT3 among others [17–19].

A number of evidence indicate a role of p27 in cancer. Results from studies performed in different human cancers showed a strong correlation between low p27 levels and a worse prognosis of the affected patients [20]. Moreover, cytoplasmatic localization of p27 also correlates with malignancy [21,22]. Taking into account the role of p27 as a transcriptional regulator it has to be considered that the decrease of p27 can be involved in tumorigenesis by deregulating the transcriptional programs under its control. This consideration implies that for a better understanding of the role of p27 in tumor development the transcriptional programs under its regulation must be known. A previous ChIP on chip study revealed the association of p27 with a number of gene promoters [14]. Interestingly, these p27-target genes (p27-TGs) are involved in relevant cellular functions as cell cycle, respiration, translation and mRNA processing and splicing. Expression of p27-TGs was subsequently studied by microarrays in mouse embryonic fibroblasts (MEFs) from control and p27-deficient animals. Results from these studies revealed that p27 mostly behaves as a transcriptional co-repressor [14]. Moreover, a correlation of over-expression of p27-TGs and poor survival could be established in different types of cancer [14].

The ChIP on chip analysis is conceptually limited to identifying the interactions of a given protein with gene promoters. Thus, the putative transcriptional regulatory role of p27 through its association with chromatin regions other than the promoters was not addressed. On considering the relevance of the role of p27 as a transcriptional regulator we aimed to extend these previous studies by identifying all the p27-binding sites (p27-BSs) in the chromatin of MEFs to better define the putative transcriptional programs regulated by this protein. To this purpose we performed ChIP sequencing analysis (ChIP-seq) on quiescent MEFs. Results revealed that most of the p27-BSs are located at distal intergenic regions. Analysis of the genes annotated to these p27-BSs has allowed the identification of relevant transcriptional programs regulated by this protein.

## Material and methods

### Cell cultures

HCT116 cells were cultured in Dulbecco's modified Eagle's medium (DMEM)–HAM F12 (1:1). MEFs (p27^WT^ and p27^-/-^) were generated with the approval of “Comitè Ètic d’Experimentació animal” from the University of Barcelona. Euthanasia of the animals was performed by cervical dislocation. C2C12 cells and MEFs were cultured in Dulbecco's modified Eagle Medium (DMEM) supplemented with 10% fetal bovine serum (Biological Industries), 2 mM L-Glutamine (Merck), 1% non-essential amino acids (Biological Industries), 50 U/ml penicillin and 50 μg/ml streptomycin (Biological Industries). MEFs also included in the medium 1 mM pyruvic acid (Sigma). Prior to synchronization MEFs were cultured up to 90% confluence and to make them quiescent they were maintained for 72 h in the medium in the absence of serum. Cell synchronization was monitored by flow cytometry of propidium iodide-stained cells.

### ChIP

Quiescent MEFs were used for ChIP with anti-p27 antibodies (Santa Cruz SC528). ChIP assay was performed as previously described [23]. Briefly, cells were lysed and chromatin from cross-linked cells was sonicated. Chromatin was incubated with 5 μg of anti-p27 in RIPA buffer (50 mM Tris−HCl pH 7.5, 150 mM NaCl, 1% NP-40, 0.5% Sodium deoxycholate, 0.1% SDS, 1 mM EDTA, 1 mM DTT, 1 mM PMSF, 0.1 mM Na_3_VO_4_, 0.5 μg/μl aprotinin, 10 μg/μl leupeptin) adding 20 μl of Magna ChIP Protein A magnetic beads (Millipore). Samples were incubated in rotation overnight at 4°C. Beads were washed with low salt buffer, high salt buffer, LiCl buffer and TE buffer. Subsequent elution and purification of the immunoprecipitated DNA-proteins complexes was performed using the IPure kit (Diagenode) according to manufacturer’s protocol. The association of p27 to their respective binding sites of target genes was performed by qPCR using the primers listed in S1 Table. ChIP data were quantified using the comparative delta Ct method [24]. Chip data from wt cells were calculated independently of the data from p27^-/-^ cells. Specifically, for all chromatin immunoprecipitations, IP and control (no antibody) values were normalized by their input and fold changes relative to control were determined using the ΔΔCt method; a mean fold change (2^-ΔΔCtAVE^) value along with a s.e.m. (abs(((2^-ΔΔCtAVE^ x 2^-ΔΔCtSEM^)—(2^-ΔΔCtAVE^ / 2^-ΔΔCtSEM^)) / 2)) value were determined.

### ChIP–seq

ChIP was performed in quiescent p27^WT^ MEFs as described above. After DNA isolation, ChIP samples, together with the input, were sequenced. Libraries were prepared using the NEBNext® ChIP-Seq Library Prep Reagent Set for Illumina® kit (ref. E6200S) according to the manufacturer's protocol. Briefly, 10 ng of input and ChIP enriched DNA were subjected to end repair, addition of “A” bases to 3′ ends and ligation of PE adapters. All purification steps were performed using Qiagen PCR purification columns (refs. 50928106 and 50928006). Library size selection was done with 2% low-range agarose gels. Library amplification was performed by PCR on the size selected fragments. Final libraries were analyzed using Agilent DNA 1000 chip to estimate the quantity and check size distribution, and were then quantified by qPCR using the KAPA Library Quantification Kit (ref. KK4835, KapaBiosystems) prior to amplification with Illumina’s cBot. Sequencing was done on the HiSeq2000, Single Read, 50 nts.

### ChIP-seq data analysis

Illumina pipeline analyzed short reads were uniquely aligned allowing at best two mismatches to the UCSC (The Genome Sequencing Consortium) mouse genome version mm9, using the program BOWTIE [25]. Peak caller algorithm MACS (version 1.4) was used to determine enriched peak region with parameters:—nomodel,—tsize = 46,—bw = 300. Shift size were determined using Pyicos [26] strand correlation method. Enriched peaks were annotated to nearest EnsEMBL [27] gene (EnsEMBL Biomart version 54) using Bioconductor package ChIPpeakAnno [28]. Distribution of enriched reads along the genomes and transcription start site (TSS) of RefSeq genes were determined using CEAS [29]. ChIP-seq data are available in the ArrayExpress database (www.ebi.ac.uk/arrayexpress) under accession number E-MTAB-5105.

### De novo motif discovery

To determine the over-represented short sequence motifs in enriched peaks, we used total 100nt sequences (from peak summit, 50bp down and 50bp up). We used top 1000 peaks (based on fold change) for finding motifs using both WEEDER [30] and MEME [31] programs.

### Putative transcription factor binding

Possible occurrence of TF motifs in peak regions (150bp around peak summit), or in target gene promoters were predicted with STORM algorithm [32] with a p-value cutoff determined based on the size of the input sequence as (1/100 x sequence-size), and using position frequency matrices (PFM) from TRANSFAC database (professional version release 2009.4).

### Co-occurrence and enrichment of transcription factors around enriched peaks

Possible occurrence of TF motifs in peak regions (150bp around peak summit) and overrepresentation of these TFs is based on the tool Pscan [33] with parameter: mixed background mm9, TRANSFAC matrix. Pscan evaluates local enrichment, comparing with a t-test mean and standard deviation of the score of the best matching oligonucleotides in the input regions to mean and standard deviation of the best match in the genomic regions flanking the input ones. Local enrichment can be used to identify motifs with significant preference for binding within the regions, that is, the motif corresponding to the ChIP’ed TF, as well as other TFs likely to interact with it and binding in its neighborhood. Local enrichment *P*-value (L.PV): describing whether the motif is over- or underrepresented in the 150 bp input regions with respect to the genomic regions flanking them, indicating whether the motif is over- represented.

### Functional enrichment analysis

Functional annotation of target genes is based on Gene Ontology (GO) [34] Consortium, 2000; (http://www.geneontology.org) as extracted from EnsEMBL [27]. Accordingly, all genes are classified into the ontology category biological process. Enrichment analysis was performed using the public DAVID tools (Database for Annotation, visualization and integrated discovery v 6.7) (http://david.abcc.ncifcrf.gov)[35].

### RNA extraction, reverse transcription-PCR (RT-PCR) and quantitative PCR (qPCR) for gene expression analysis

Total RNA from cells was extracted using High Pure RNA Isolation kit (Roche). cDNA was obtained from 1 μg of RNA using SuperScript VILO cDNA synthesis (Invitrogen) according to manufacturer’s instructions. Gene expression was analyzed by real-time PCR, using Express SYBR GreenER qPCR supermix (Invitrogen), corrected by GADPH expression and expressed as relative units. Primer sequences used for qPCR assessment of mRNA levels of target genes are listed in S2 Table.

### Immunoprecipitation and immunobloting analysis

Proliferating MEFs and C2C12 cells were scraped and washed twice with PBS. Pellets were lysed in 1 ml of IP buffer (PBS containing 0.5% Triton X-100, 1 mM EDTA, 100 μM sodium orthovanadate, 0.25 mM PMSF, complete protease inhibitor mixture by Roche Applied Science, and 1/25 vol of DNAse I (Sigma). Cell lysates were incubated on ice for 30 min and then centrifuged at 3000 rpm at 4°C for 5 min. After centrifugation, samples were quantified using Lowry method [36]. 1 mg of total protein was incubated overnight at 4°C with 2 μg of anti-p27 (Santa Cruz SC528). Samples were incubated with A-Dynabeads (Invitrogen) for 2 h and the immunocomplexes were extensively washed with IP buffer, subsequently eluted with 0.1 M Citrate pH 2.5 and boiled at 100°C in Laemmli buffer for Immunobloting analysis. As a control, lysates were incubated with irrelevant rabbit IgG. Antibodies used for Immunoblotting were: anti-p27 (BD Transduction Laboratories 610242; 1:1000), anti-Pax5 (Santa Cruz SC1974; 1:500), anti-MyoD (Santa Cruz SC760; 1:500) and anti-AHR (ENZO life Sciences BML-SA210-0100; 1:2000).

### Cell adhesion analysis

To examine the behavior of p27 deficient cells in terms of adhesion we used the *Roche xCELLigence real-time cell analysis system*. This system measures the area that is occupied by the cell on real-time which represents itself as impedance. During the time the cell attaches on the culture plate the area occupied increases and this causes an impedance of the current. Cell-index values reflecting impedance changes are automatically and continuously recorded by the RTCA DP instrument. wt and p27^-/-^ MEFs were trypsinized and the same number of cells were plated on xCELLigence plates. Normalized cell index data obtained few hours after cell plating are plotted over time axis. Slope of this graph is interpreted as substrate adhesion capacity of the cells.

### β-Galactosidase and luciferase assays

For luciferase assays the DNA sequences corresponding to the p27-BSs annotated to Mef2c, SOX6 and Shox2 (S3 Table) were inserted into pGL3 reporter vectors. Then, HCT116 -WT or HCT116 -p27^-/-^ cells were co-transfected with CMV-βGal vector and an empty vector, and luciferase vectors containing the selected p27BSs regions, using Lipofectamine 2000 reagent (Invitrogen) following the manufacturer’s protocol. Cells were ONPG (Sigma) and read at 405 nm wavelength. Luciferase assays were performed using the Dual-Luciferase reagent (Promega) and a Lumat LB9501 luminometer (Berthold).

### Statistical analysis

GraphPad Prism 5.01 (GraphPad Software, San Diego, CA, USA) was used for data analysis. The Student’s t-test was applied to determine significant differences between groups. In these analyses P-values <0.05 were considered to be significant. At least three independent samples were analyzed in each experiment.

## Results

### p27 ChiP-seq in mouse embryonic fibroblasts identifies novel target genes

**Fig 1:**
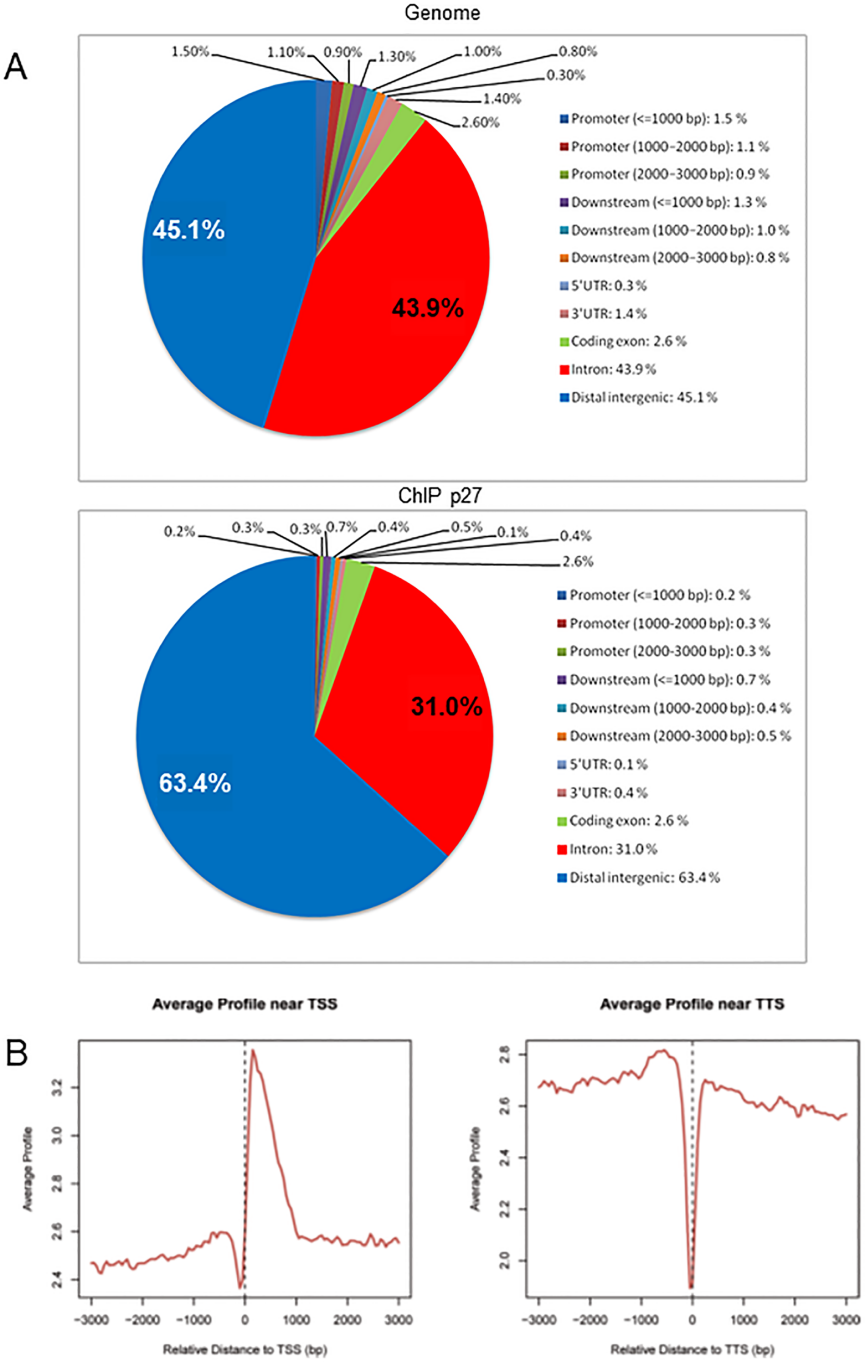
Genomic distribution of p27 binding sites along the chromatin. (A) Distribution of different chromatin regions overall genome in MEFs (upper panel) and of the p27 binding sites (Bottom panel) obtained by ChIP-seq experiments. (B) Distribution of p27 binding sites around the transcription starting sites (TSS) and transcription terminal sites (TTS) represented as average profile.

We performed ChIP-seq analysis using an anti-p27 antibody (Santa Cruz SC528). The fragmented chromatin (average size 500 bp) was immunoprecipitated and the DNA deep-sequenced alongside with the non immunoprecipitated input. Using a threshold p-value<10^−6^ and a false discovery rate (FDR) <20% in the immunoprecipitation vs. input comparison, we identified 1839 sites bound by p27 (S1 Fig). These peaks were annotated to the most proximal transcriptional start sites (TSSs) of genes giving 1417 different genes, thus, indicating that some genes have more than one proximal p27-binding site (p27-BS). In order to test whether p27 has a differential preference of interaction over the different chromatin regions, we compared their presence on specific chromatin regions namely: promoters (till 3000 bp upstream of TSSs), proximal downstream regions (till 3000 bp downstream of the transcriptional termination sites (TTSs)), 5’-UTR regions, 3’-UTR regions, coding exons, introns and distal intergenic regions (Fig 1A). We observed that the frequency of distribution of p27 peaks over most of the regions is significantly lower that the distribution of genomic sequences. The exceptions are the interaction with coding exon sequences that being 2.6% is similar to genomic distribution and the association with distal intergenic regions that approximately include the 63.4% of the peaks, significantly higher than the distribution of genomic sequences (Fig 1A). Altogether, these results indicate that the association of p27 with chromatin may have a most significant role at distal intergenic domains but also at intronic regions. As shown in Fig 1B (left panel) the distribution of p27-BSs around the TSS presents a peak just after TSS (till the first 1000 pb) and a decrease just before. Differently, the profile of p27-BSs in the TTS region shows a strong decrease just at the TTS (Fig 1B, right panel).

**Fig 2:**
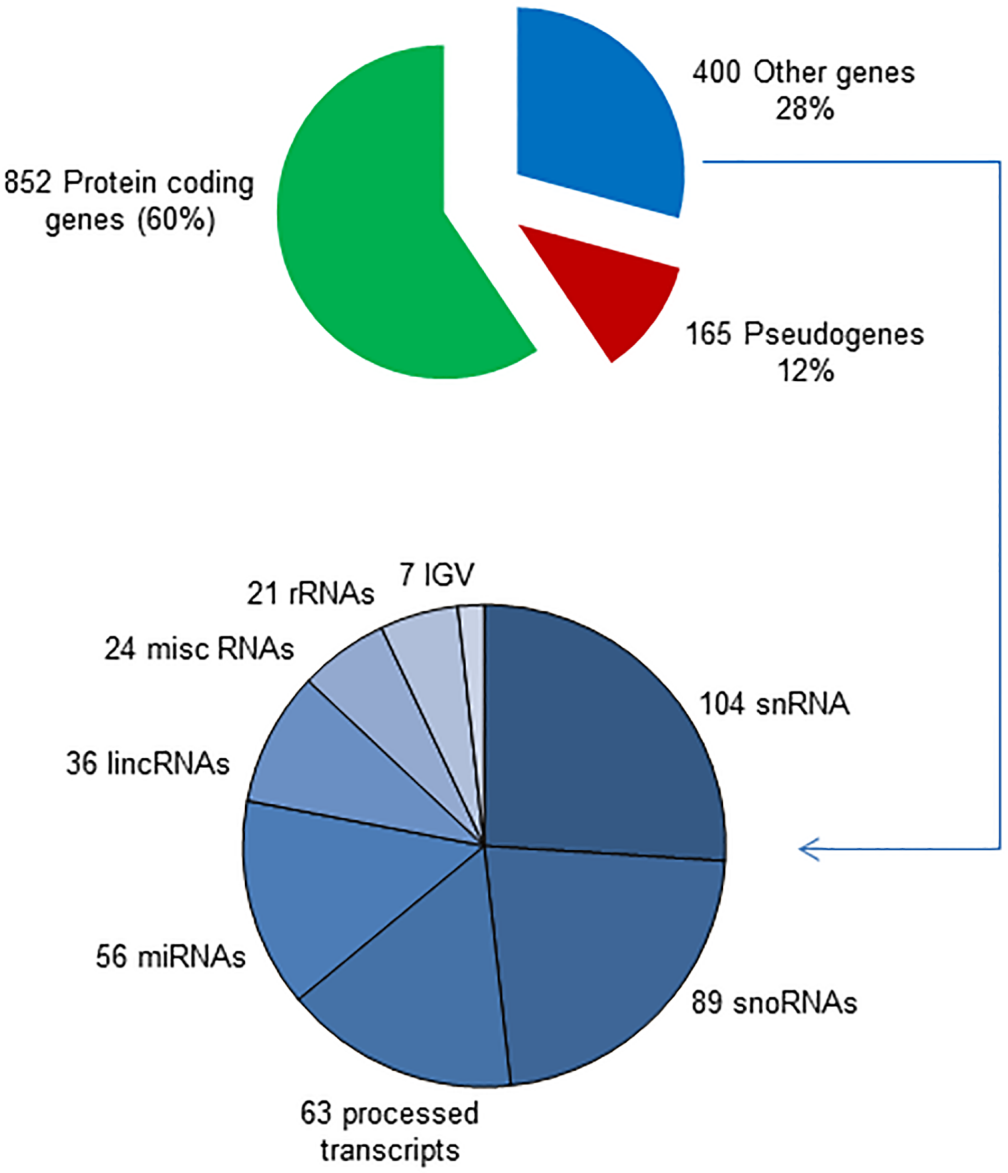
Gene biotype distribution of genes with p27 binding sites in their vicinity. Distribution of the 1447 annotated genes containing at least a p27-BS in their vicinity, according to their biotype (upper panel). The distribution of genes other than those encoding for proteins protein-coding is shown in the bottom panel.

**Fig 3:**
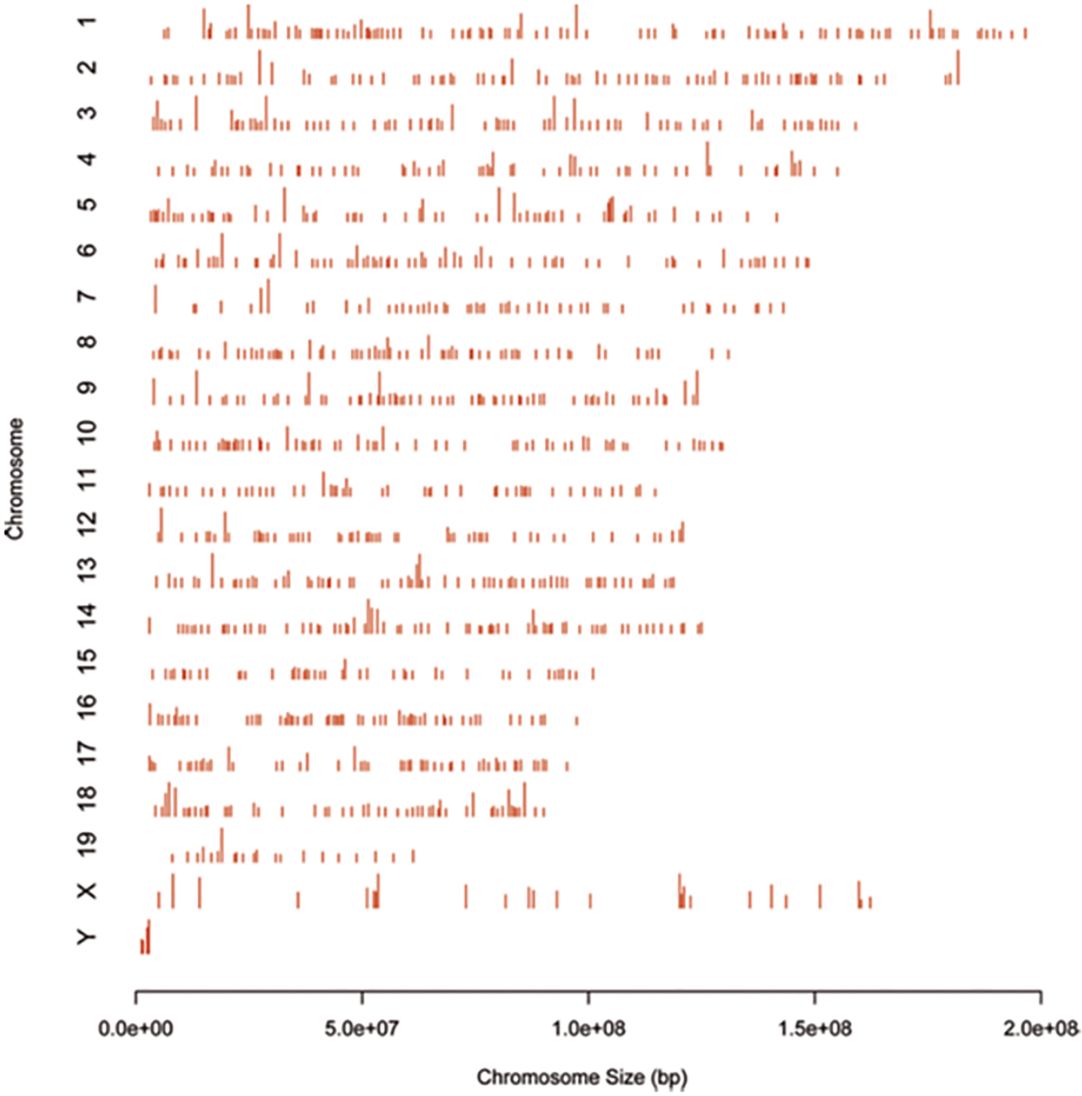
Distribution of p27 binding sites over the chromosomes. The graph represents the distribution of p27-binding sites over the chromosomes. Bars represent significant p27 peaks. Length of the bar represents peak strength.

These 1417 genes were subsequently classified according to the biotype. Results revealed that approximately the 60% of them (852 genes) corresponded to protein coding genes, the 12% to pseudogenes and the other (28%) include immunoglobulin genes and different types of non-coding RNAs (Fig 2). These observations suggest that p27 can have a putative role not only in the regulation of protein coding genes but also in the expression of pseudogenes and a variety of non-coding RNAs. The distribution of the p27-BSs over chromosomes is shown in Fig 3. As it can be seen p27 associates with all chromosomes but the binding is minimal in chromosome Y probably due to low number of genes in this chromosome.

**Fig 4:**
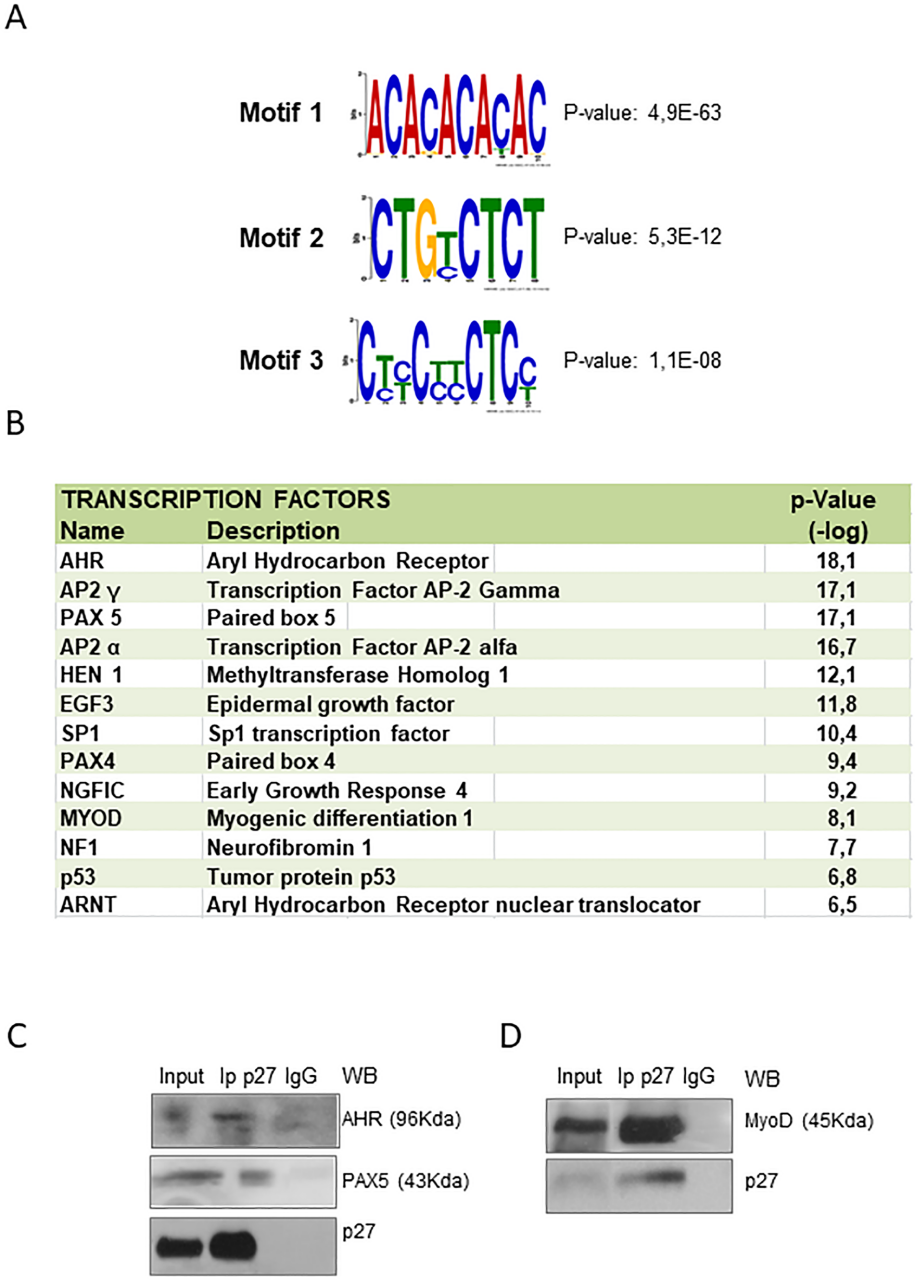
Enriched sequences in the p27 binding sites and transcription factors that putatively can associate to these sequences. (A) Enriched short sequence motifs in p27 peaks throughout the genome. Short sequence motifs were determined using both WEEDER and MEME program. (B) Putative transcription factors with matching motifs in p27 peak regions. (C) MEFs were immunoprecipitated with anti-p27 and immunoprecipitates were analysed by western blot using anti-AHR, anti-PAX5 and anti-p27. (D) C2C12 cells were immunoprecipitated with anti-p27 and immunoprecipitates were analysed by western blot with aniti-p27 and anti-MyoD. Immunoprecipitation with a non-specific IgG was used as a control. The presence of these proteins in cell lysates are also shown (input).

To determine whether DNA sequences associated with p27 contained overrepresented motifs, these DNA sequences were studied using the MEME program [31]. These analyses revealed three overrepresented sequences. These were: ACACACACAC tandem repeat (p-value: 4.9e-063), CTGx(T/C)CTCT (p-value: 5.3e-012) and Cx(T/C)x(T/C)Cx(T/C)x(T/C)CTCTx(T/C) (p-value: 1.1e-08) (Fig 4A). Comparison of these sequences with known transcription factor (TF) binding sequences using the MEME program revealed that the sequences that are overrepresented in the p27 peaks showed strong similarity to consensus binding sequences of some TFs. Some of them were AHR, Ap2α and Ap2γ, Pax5 and Pax4, and MyoD, among others (Fig 4B). Taking into account that p27 does not have a defined DNA binding motif in its structure, the association of p27 with chromatin would, probably, be mediated by interacting with these specific TFs. This association would depend on the chromatin region but also of specific intracellular signals triggered by regulatory factors acting on the cells. To test the putative interaction of p27 with several of these TFs we performed IP experiments using an anti-p27 antibody (see methods) and checking the TF association by western blot. As shown in Fig 4C and 4D, all the tested TFs (AHR, Pax5 and MyoD) were found to associate with p27, suggesting a collaboration between p27 and these specific TFs in transcriptional regulation.

### GO analysis of protein-coding genes

**Fig 5:**
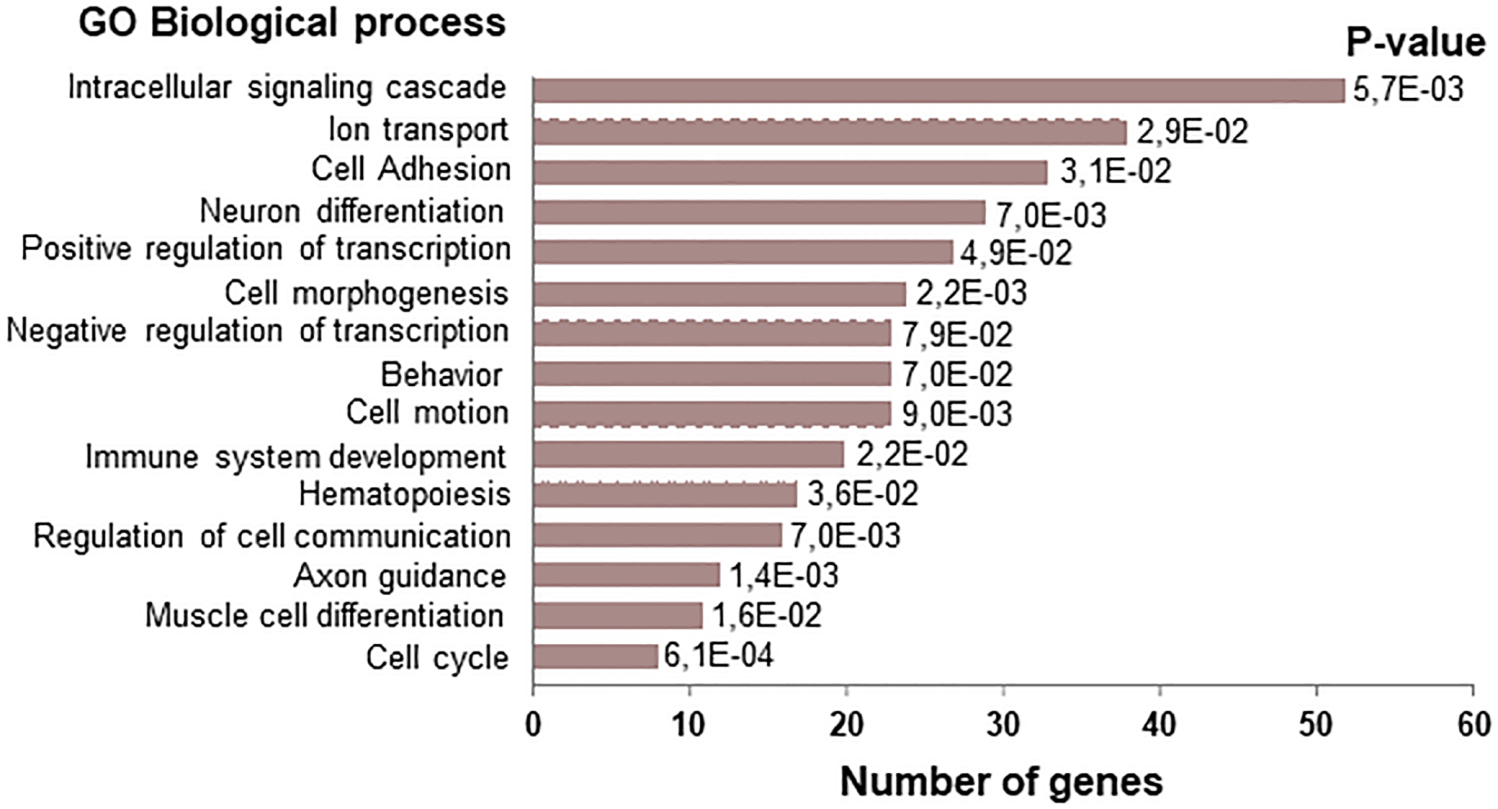
Gene Ontology analysis of genes with p27 binding sites. Gene Ontology (GO) analysis of the protein coding genes with p27 binding sites in their proximity is shown. The Database for Annotation, Visualization and Integrated Discovery (DAVID) program was used to determine biological processes enriched in the 852 protein coding genes.

The 852 protein coding genes that contained a p27 peak at its vicinity (S2 Fig), were subjected to Gene Ontology (GO) analysis using the DAVID Bioinformatics program [35]. The most enriched GO terms (Biological process) included: intracellular signaling cascade (52 genes), ion transport (39 genes), cell adhesion (32 genes), neuron differentiation (28 genes), positive regulation of transcription (27 genes), cell morphogenesis (25 genes), negative regulation of transcription (24 genes), behavior (24 genes), cell motion (24 genes), immune system development (20 genes), hematopoiesis (17 genes), regulation of cell communication (16 genes), axon guidance (12 genes), muscle cell differentiation (11 genes) and cell cycle (7 genes) (Fig 5).

**Fig 6:**
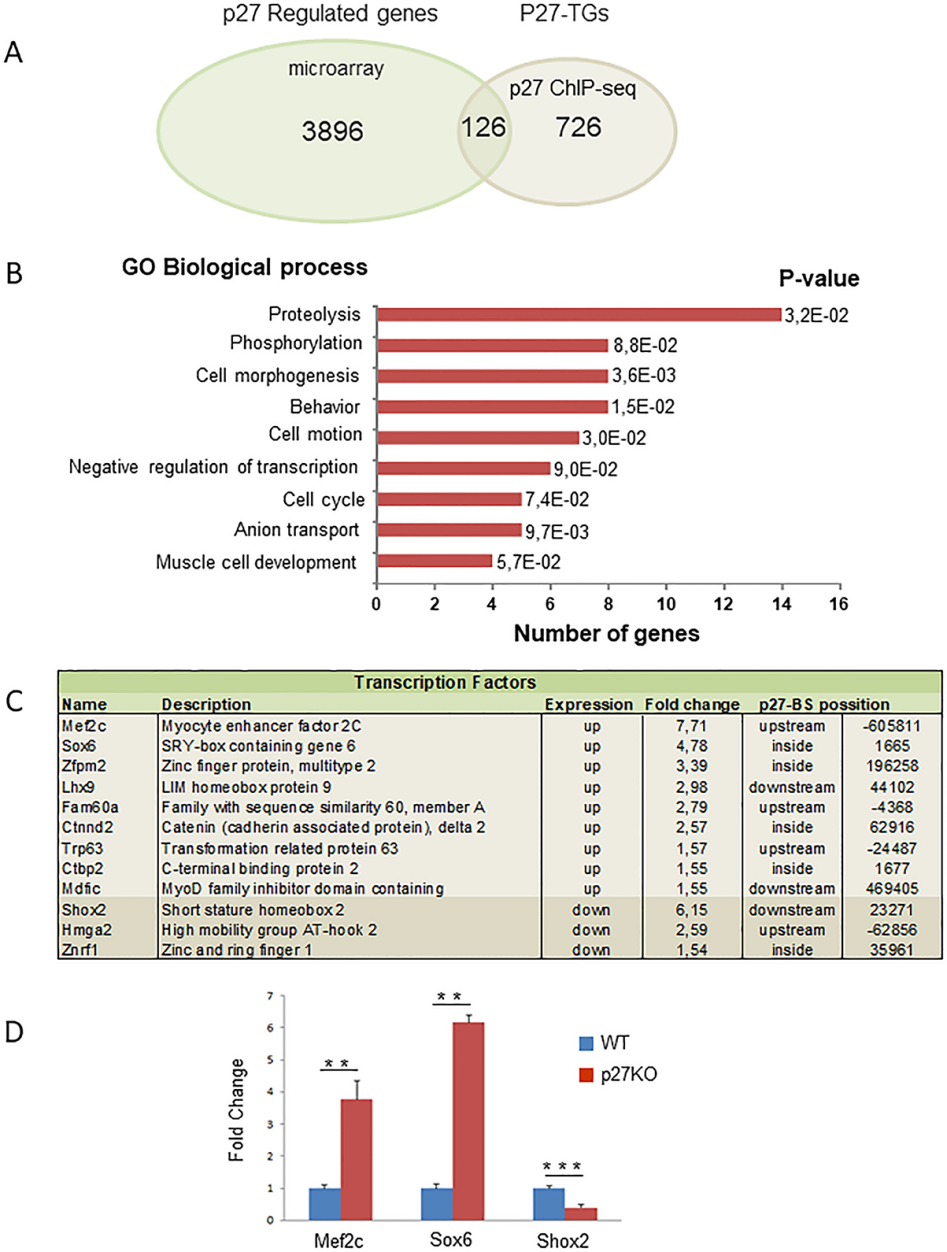
Identification of genes showing p27 binding sites in their vicinity and that are differentially expressed in p27 deficient MEFs. (A) Venn diagram representing the number of protein coding genes differentially expressed in cells lacking p27 and having p27 binding sites in their vicinity. (B) GO analysis of the 126 identified common genes. (C) List of transcription factors included in these 126 common genes. The expression of these transcription factors in p27 knock out MEFs is represented as the fold change versus control cells. The localization of the p27 binding site respect to the transcription starting site is also indicated. (D) The effect of p27 silencing on transcription was determined by luciferase assays using a pGL3 vector including the p27-BSs annotated to Mefc2, Sox6 and Shox2. P27WT and p27^-/-^ cells were co-transfected with the pGL3 vector and the CMV-βGal vector. Luciferase assay were performed as described under material and methods. Results are the mean value ± SD of three independent experiments. Statistical analyses were performed using the t-student’s test. (**) *P*<0.01 and (***) *P*<0.001.

Expression microarray analysis showed that 4022 genes were differentially expressed in p27^-/—^MEFs [16]. In MEFs, we have detected 1417 p27-BSs being 852 of them annotated to protein coding genes. On comparing these 852 genes with those differentially expressed in p27^-/-^ MEFs we observed that only 126 of them were expressed in quiescent p27^-/-^ cells (Fig 6A), being 62 down-regulated and 64 up-regulated. Using the DAVID program we also looked for the GO terms enriched in these 126 p27-TGs. Proteolysis, phosphorylation, cell morphogenesis, behavior, cell motion, negative regulation of transcription, cell cycle, anion transport and muscle cell development were the GO terms mostly enriched in this group of genes (Fig 6B). Most of these terms were also observed in the biological processes enriched in the p27 ChIP-seq (Fig 5). Altogether, these results indicate that in quiescent p27^-/-^ cells only a relatively reduced number of the putative p27-TGs (126 genes out of 852) (~15%) were differentially expressed, despite the total number of differentially expressed genes was high. These results suggest that the role of most of the putative p27-TGs might be relevant in non-quiescent cells, for instance in proliferating cells, or in other cellular types.

The evidence that we have been not able to detect putative p27-BSs in the proximity of most of the genes differentially expressed in quiescent p27^-/-^ cells [16] suggested that p27 could indirectly regulate the expression of most of these genes by controlling the expression of TFs. To explore this possibility, TFCONES database [37] was used to identify genes encoding TFs among these 126 p27-TGs. Results revealed that 12 genes containing p27-BSs and differentially expressed in p27^-/-^ cells encode TFs (Fig 6C). To demonstrate that actually p27 regulates the expression of these TFs trough the annotated p27-BSs, we performed luciferase assays using a luciferase reporter gene including these specific p27-BSs. As shown in Fig 6D, when using the p27-BSs annotated to Mefc2 or to Sox6, luciferase expression was increased in the p27^-/-^ cells. In contrast, in the case of the sequences annotated to Shox2 luciferase expression was significantly downregulated in cells lacking p27. These results reveal that p27 can regulate specific transcriptional programs not only by directly controlling the expression of genes included in these programs but also by the direct control of TF expression. In such a way, the transcription response after p27 decrease in cells would be significantly amplified.

### p27 regulates the expression of genes involved in signal transduction

**Fig 7:**
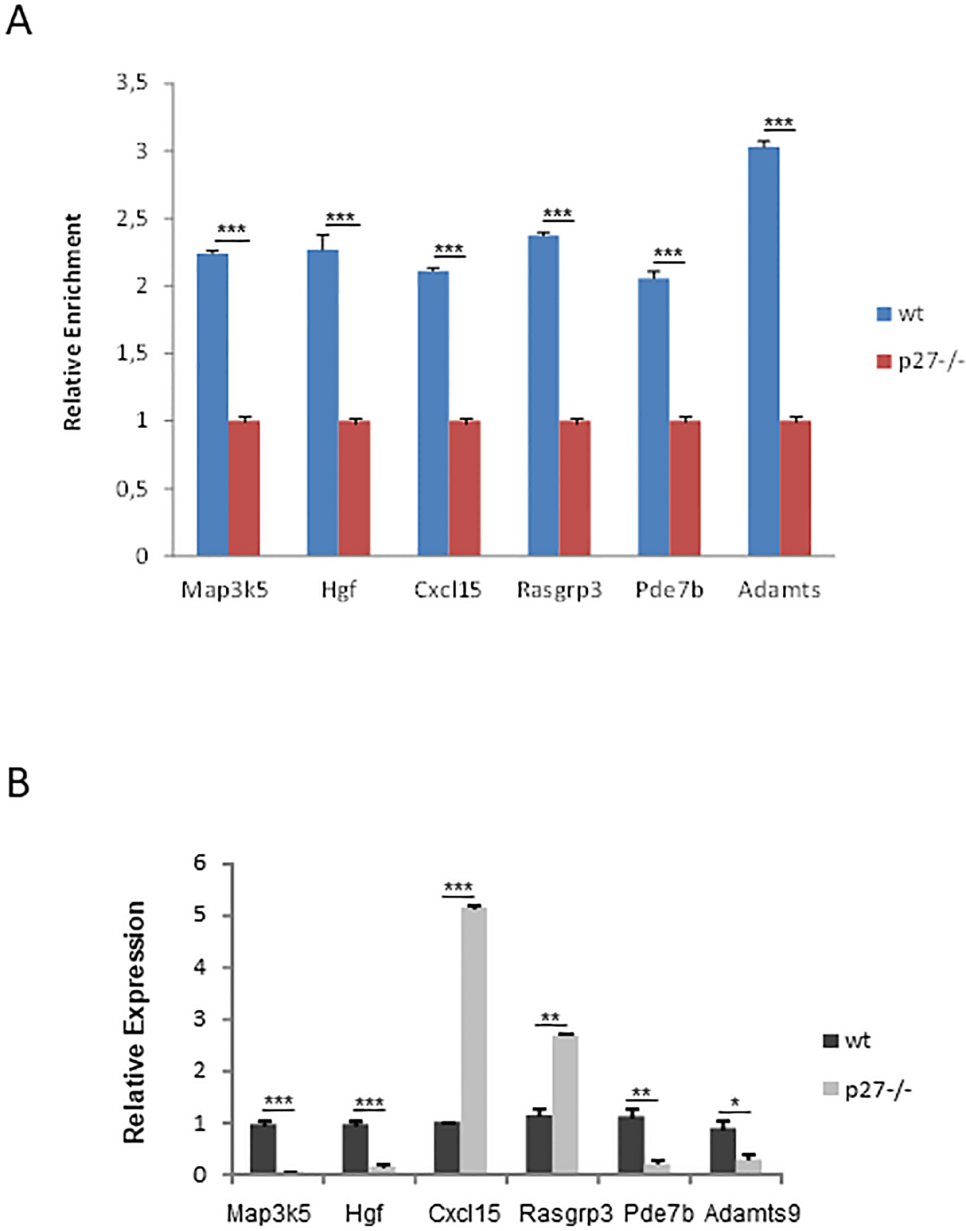
p27 regulates the expression of cell signaling genes. (A) Analysis of the association of p27 with specific p27 binding sites of the detailed cell signaling genes. Interaction was determined by ChIP followed by qPCR using specific primers in p27^WT^ and p27^-/-^ MEFs. ChIP data were quantified using the comparative delta Ct method (see methods). Ct values resulting from p27^WT^ and p27^-/-^ MEFs were normalized both versus the input and subsequently versus no-antibody control. Results are expressed as relative enrichment of values from p27^WT^ versus those from p27^-/-^. Results are the mean value ± SD of three independent experiments. Statistical analyses were performed using Prism 6.0 software (GraphPad Software, Inc., Ca,USA) and the P-values were calculated using two-tailed *t*- test. (***) *P* <0.001. (B) mRNA levels of the indicated cell signaling genes in p27 deficient MEFs versus control as determined by qPCR. Expression levels were normalized according to GAPDH gene expression. Results are presented as relative expression and represent the mean value ± SD of three independent experiments. Statistical analyses were performed using the *t*-student’s test. (*) *P* <0.05, (**) *P* <0.01 and (***) *P* <0.001.

As it can be seen in Fig 5 one of the more represented transcriptional program putatively regulated by p27 is the intracellular signaling cascade. In addition to the 52 genes included in this group by the DAVID program, the manual annotation of the genes identified by the ChIP-seq analysis, has allowed to increase the number of genes of this group until 107 (S3 Fig). The list of these genes includes ligands, receptors, G-proteins, GEFs, GAPS, kinases, phosphatases and cyclases. Thus, we aimed to validate the interaction of p27 with the specific regions described in the ChIP-seq analysis for a group of these genes. Validation was performed by ChIP using an anti-p27 antibody (see methods) followed by qPCR using primers specific for these sequences. As a control we performed ChIP in p27^-/-^ MEFs. As shown in Fig 7A, p27 specifically associates with the p27-BSs corresponding to Map3k5, Hgf, Cccl15, Rasgrp3, Pde7b and Adamts9. ChIP experiments using non-specific IgG as controls revealed similar results (data not shown). We also observed that all these genes are actually regulated by p27 because the mRNA levels corresponding to these genes are altered in MEFS lacking p27. As shown in Fig 7B the expression of Map3k5, Hgf, Pde7b and Adamts9 was decreased in MEFs p27^-/-^ whereas those of Cxcl15 and Rasgrp3 were increased.

### p27 regulates cell adhesion

**Fig 8:**
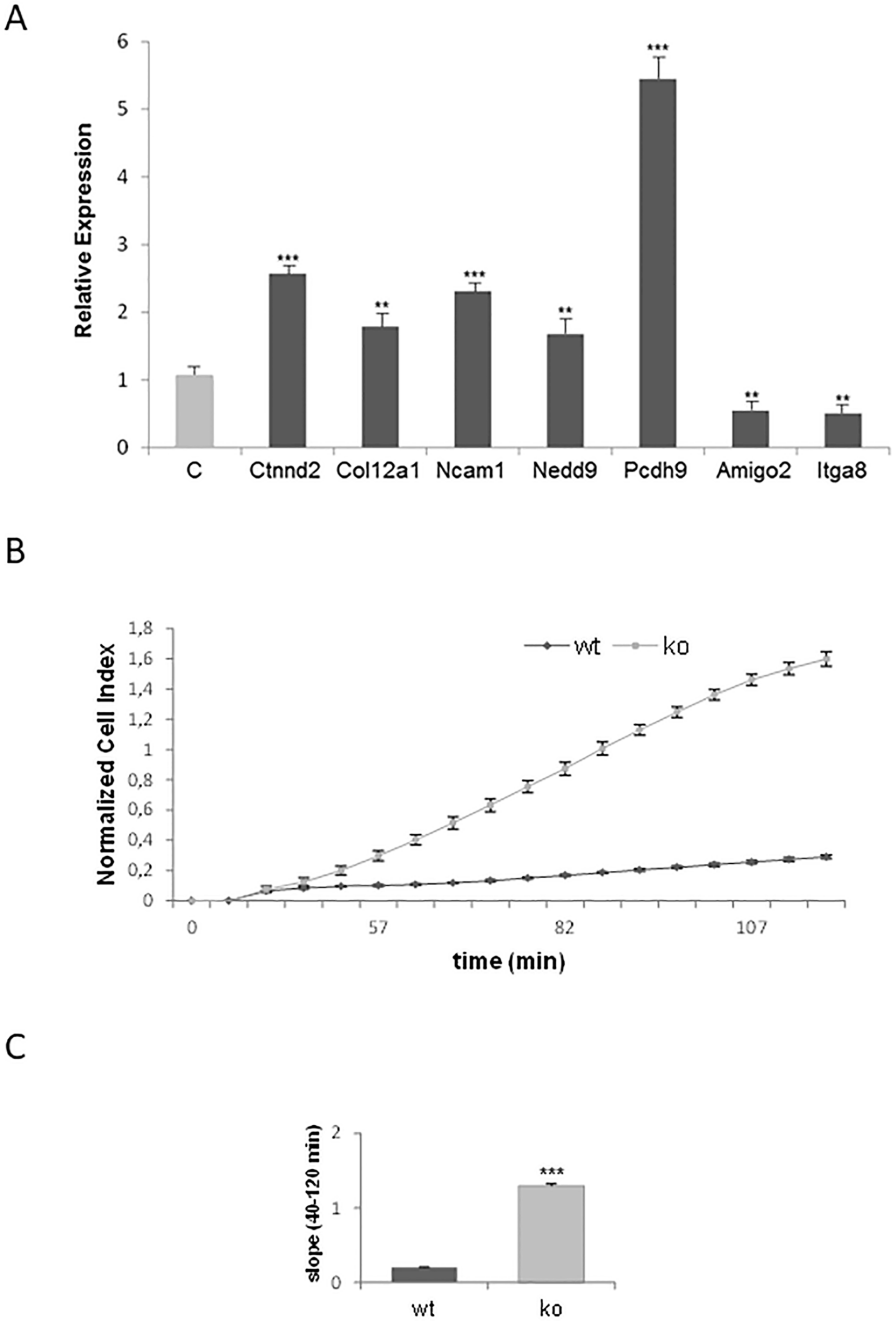
p27 regulates cell adhesion. (A) mRNA levels of the indicated cell adhesion genes in p27 deficient MEFs versus control (C) as determined by qPCR. mRNA levels are expressed as relative to control cells and represent the mean value ± SD of three independent experiments. Statistical analyses were performed using the *t*-student’s test. (**) *P* <0.01 and (***) *P* <0.001. (B) Cell adhesion analyses of p27^WT^ and p27^-/-^ MEFs. The same number of cells was plated on xCELLigence plates. Normalized cell index data obtained at different hours after plating are plotted over time axis. (C) Slope of the graphic in (B) represented the substrate adhesion capacity of the cells. Values represent the mean value ± SD of three independent experiments. Statistical analyses were performed using the *t*-student’s test. (***) *P* <0.001.

Cell adhesion was also a transcriptional program regulated by p27. ChIP-seq analysis revealed that 32 genes belonging to this group were annotated. Interestingly, cell adhesion was the most significantly enriched term in GO analysis and Kegg pathways that were altered in p27^-/-^ cells [16]. Thus, we aimed to determine by qPCR whether the expression of a number of genes found in the ChIP-seq analysis was altered in p27 deficient MEFs. As shown in Fig 8A a group of genes including: Ctnnd2, Col12a1, Ncam1, Nedd9, and Pcdh9, were up-regulated in p27^-/-^ cells whereas the genes Amigo2 and Itga8 were down-regulated. These data supports that p27 is involved in the transcriptional regulation of genes involved in cell adhesion and that have p27-BSs in their vicinity. To examine the behavior of p27 deficient cells in terms of adhesion we used the *Roche xCELLigence real-time cell analysis system*. Thus, p27^wt^ and p27^-/-^ MEFs were trypsinized and the same number of cells was plated on xCELLigence plates. Normalized cell index data obtained few hours after cell plating were plotted over time axis. As observed in Fig 8B, at different times post-plating, p27^-/-^ MEFs had greater adhesion capacity to culture plates than control cells. Slope of this graph is interpreted as substrate adhesion capacity (Fig 8C).

## Discussion

In human tumors low levels of p27 are associated to increased malignancy and a worse outcome. As p27 has been identified as a transcriptional regulator, identifying the transcriptional programs regulated by p27 will help deciphering the role of this protein in tumor development. Specifically, it has been postulated that reduction of p27 in tumors would deregulate the expression of p27-TGs and in such a way facilitating tumor progression.

ChIP-seq analysis performed in quiescent MEFs allowed the identification of 1417 genes displaying p27-BSs in their vicinity and annotated as putative p27-TGs. This study revealed several interesting observations:

1) Only a small number of p27-BSs were on gene promoters. In contrast, most of them were in distal intergenic or intronic regions. These results expand the information on the identification of p27-BSs obtained by ChIP on chip in NIH3T3 cells in which only the association of p27 with gene promoters was analyzed [14]. Thus, results reported here reveal, for the first time, that the role of p27 in the regulation of transcription is mainly produced by associating to intergenic chromatin regions distal from the promoters or in intronic domains being the association with gene promoters more reduced. On comparing data from these two studies revealed a reduced coincidence in the identified genes. These results indicate that these two experimental approaches, ChIP-seq and ChIP on chip provide complementary information about the putative p27-BSs.

2) The analysis of the p27-BS sequences indicates that they are enriched by motifs encoding a number of TFs that could mediate the transcriptional regulatory role of p27. The most significant TFs that putatively can interact with the p27-BSs include: AHR, AP2γ and AP2α, Pax4 and Pax5, HEN1, EGF3, SP1, NGFIC and MyoD (Fig 4B). IP experiments revealed that actually p27 associates with all TFs analyzed here, namely: AHR, Pax5 and MyoD suggesting that p27 can regulate transcription by associating to these specific TFs. These results increase the number of TFs able to associate to p27 revealing a high versatility of p27 for regulating transcription.

In a previous report [14] we described that the association of p27 with gene promoters was mainly mediated by its interaction with E2F4/p130 or with members of the Ets family of TFs, as for instance Ets-1. Results reported here showing that the interaction of p27 with chromatin regions other than promoters (i.e. distal intergenic or intronic regions) could be mediated by other TFs as AHR, Pax5 or MyoD revealed that p27 is able to interact with a high diversity of TFs and suggests that these different interactions might specifically occur in distinct chromatin regions.

3) A significant number of the p27-BSs are close to pseudogenes or sequences of non-coding RNAs (ncRNAs) suggesting a putative role of p27 in the regulation of ncRNAs and pseudogene expression. Due to the relevance of these types of RNAs in multiple cellular functions and their involvement in different pathologies, as for instance cancer, these data reveal a putative new role of p27 as a transcriptional regulator of these RNAs [38–41].

4) The functional classification of the 825 protein coding genes putatively regulated by p27 revealed that significant cellular functions as: Intracellular signaling, ion transport, cell adhesion, neuron differentiation and regulation of transcription can be under the regulation of p27. Specifically, we identified 107 protein coding genes involved in cell signaling that include ligands, receptors and members of most of the steps of signaling pathways. We found ligands and receptors of from significant signaling pathways as for instance the Wnt, ephrin, and BMP pathways [42–44]. Moreover, different Interleukins and interleukin receptors, involved in the inflammatory response, are also in this list. Finally, many ligands participating in different functions of the nervous system have also been observed, suggesting a clear role of p27 in processes as development of nervous system, neuron differentiation and axon guidance.

We also identified 32 protein kinases involved in different functions as for instance cell cycle (Cdks), stress response (MAPKs), cell contraction (Mylks) or glucose metabolism (HK1) that are putative p27-TGs. We report here that actually p27 is regulating the expression of some members of the cell signaling program in some cases as a repressor and in some others as a co-activator. Altogether these results reveal that the decrease of p27 observed in human tumors might deregulate a number of cell signaling pathways that could be involved in cancer progression and malignancy.

Another very interesting transcriptional program regulated by p27 is cell adhesion. This program includes genes involved in cell-cell interaction, in the different types of cell junctions, cell-extracellular matrix interaction and components of the cytoskeleton. In this case we also report that different genes of this program are actually regulated by p27 and as mentioned before p27 could act as a repressor or as a co-activator. Interestingly, in this case we additionally demonstrated that actually p27 deficient cells display an increased adherence to the culture plates indicating a role of p27 in cell adhesion. The relevance of these results remains to be explored although it appears that for instance during development when important processes needing changes in cell motility and adhesion are produced, p27 could have an important role. Moreover, the participation of p27 in cell adhesion can also have a crucial relevance in migration and metastasis. As mentioned above, a correlation between low levels of p27 and a higher malignancy in tumors has been reported. Thus, this increased adherence of p27 lacking cells might have a key role in the collective cell migration involved in metastasis observed in highly aggressive tumors. In these type of migration cells move as a group of tightly associated cells [45].

**Fig 9:**
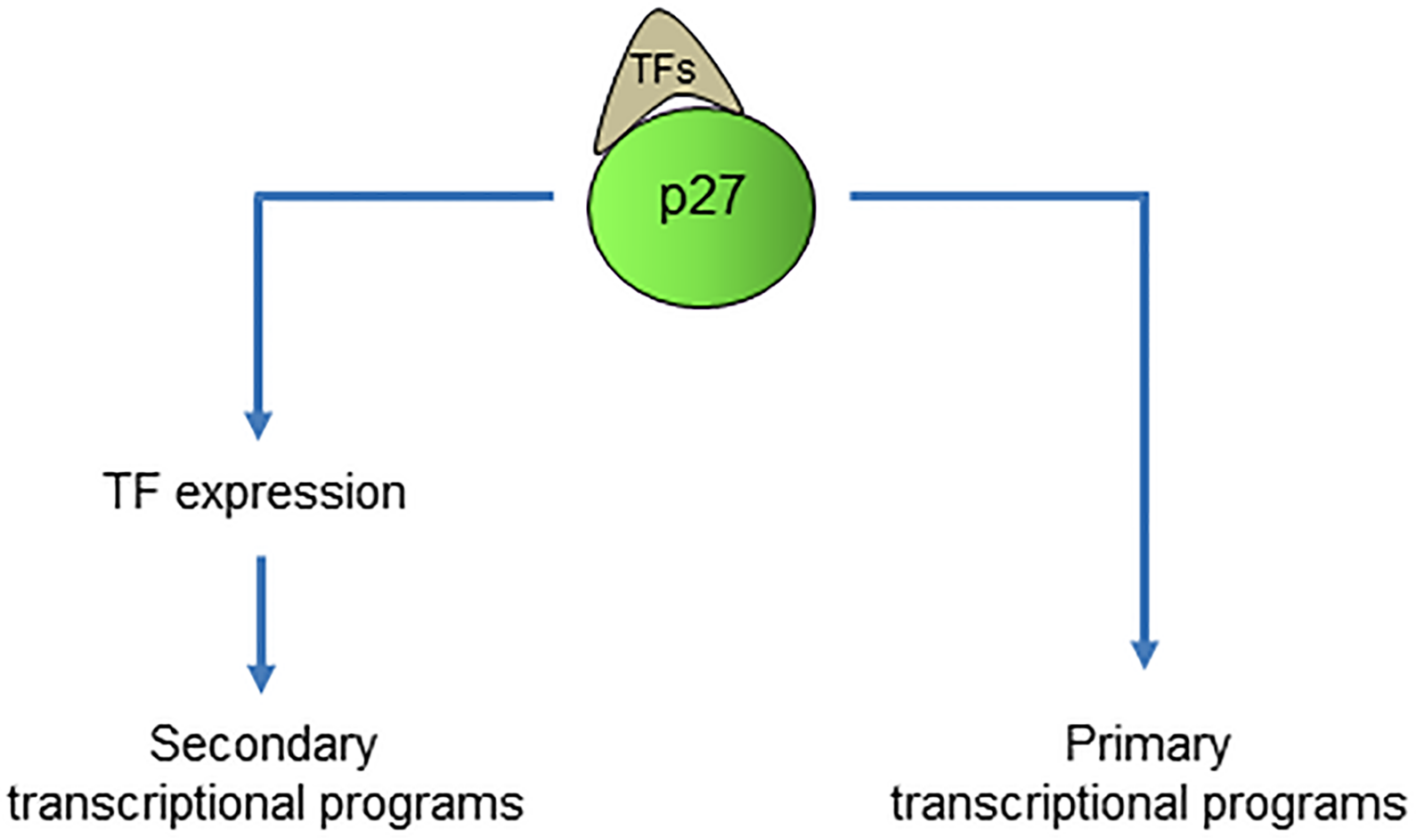
p27 regulates specific transcriptional programs. The model represents the direct role of p27 in the regulation of primary transcriptional programs putatively modulated by its association with specific transcription factors (TFs). The model also describes that by this way it is also regulating the expression of other different TFs that would be involved in the control of a secondary wave of transcriptional programs.

5) We identified 126 genes that have a p27-BS in the vicinity of the TSS and that their expression was modified in cells lacking p27. We postulate that this group of genes includes those that are directly regulated by p27 in quiescent MEFs. Probably, the rest of genes containing p27-BSs but that are not differentially expressed in p27^-/-^ MEFs could be expressed under other cellular conditions (for instance during proliferation) or perhaps in other cellular types. Interestingly, this group of 126 common genes includes 12 TFs. We postulate that these TFs, directly regulated by p27 in quiescent MEFs, could be involved in amplifying transcription leading to the expression of other different genes that could cover all those differentially expressed in p27 deficient cells as observed in the microarray experiments (Fig 9). Interestingly, among these TFs are some very relevant for the transcriptional programs regulated by p27. It merits to be mentioned Mef2c, involved in muscle differentiation but also in bone and neuronal development and that is increased more than 7 fold in p27^-/-^ cells [46]. It is also relevant the observed increase of Sox6, involved in neuron differentiation and chondrogenesis [47]. Also particularly interesting is elevated expression of p63, a member of the tumor suppressor p53 family. This family is considered to be master regulators of a plethora of important cellular functions [48]. Intriguingly, p63 plays an important role in maintaining epithelial stemness [49].

As a summary we report here the identification, by ChIP-seq, of a number of genes whose expression is regulated by p27 and that are included in transcriptional programs involved in relevant cellular functions that could be crucial for understanding the role of p27 in tumor development.

## Supporting information

S1 Fig Processed data of the p27 peaks from the ChIP-seq. (XLS)

S2 Fig List of p27 binding sites in the vicinity of protein coding genes. (PDF)

S3 Fig List of putative p27 target genes involved in cell signaling. (PDF)

S1 Table List of primers used for ChIP studies. (PDF)

S2 Table List of primers used for expression experiments. (PDF)

S3 Table List of sequences of the p27-BSs used for luciferase assays. (PDF)
