# Supplementary material for: ChIP-Seq analysis identifies p27(Kip1)-target genes involved in cell adhesion and cell signalling in mouse embryonic fibroblasts
Source: PLoS One. 2017 Nov 20;12(11):e0187891. doi: 10.1371/journal.pone.0187891 (PMC5695801; doi:10.1371/journal.pone.0187891)
Supplement: S2 Fig — (PDF) [file pone.0187891.s002.pdf]

List of p27-binding sites in the vicinity of protein coding genes

chr	start	end	-10*log10(pvalue)	insideFeature	distance to Target	Ensembl_Gene_ID	GeneSymbol
chr4	11398866	11399230	80,73	upstream	-14239	ENSMUSG00000040720	1110037F02Rik
chr14	79202193	79202528	55,48	upstream	-46792	ENSMUSG00000058997	1300010F03Rik
chr10	83468327	83468647	59,09	downstream	282717	ENSMUSG00000087651	1500009L16Rik
chr3	6712173	6712438	53,35	upstream	-91730	ENSMUSG00000069118	1700008P02Rik
chr3	6511342	6511581	59,62	downstream	109101	ENSMUSG00000069118	1700008P02Rik
chr3	48154531	48154830	69,81	downstream	258492	ENSMUSG00000037910	1700018B24Rik
chr10	38838617	38838913	60,29	overlapEnd	3503	ENSMUSG00000051736	1700025K23Rik
chr12	36969006	36969326	64,03	inside	10979	ENSMUSG00000020545	1700108M19Rik
chr12	36917821	36918201	55,94	downstream	26968	ENSMUSG00000020545	1700108M19Rik
chr10	86664335	86664692	50,60	upstream	-4389	ENSMUSG00000047129	1700113H08Rik
chr12	73631232	73631530	54,83	upstream	-6112	ENSMUSG00000034501	1810048J11Rik
chr4	25192455	25192690	51,18	downstream	16440	ENSMUSG00000040359	1810074P20Rik
chr13	63234122	63234468	57,59	inside	117595	ENSMUSG00000021458	2010111I01Rik
chr17	36290521	36290801	57,76	upstream	-1271	ENSMUSG00000038311	2410017I17Rik
chr9	58589068	58589362	51,01	inside	298	ENSMUSG00000074269	2410076I21Rik
chr3	90305382	90305751	52,47	downstream	7562	ENSMUSG00000001017	2500003M10Rik
chr12	101011933	101012272	72,87	inside	109719	ENSMUSG00000021176	2610021K21Rik
chr4	145751509	145751956	65,29	upstream	-2493	ENSMUSG00000070605	2610036A22Rik
chr12	88790318	88790645	55,04	upstream	-5351	ENSMUSG00000021041	2700073G19Rik
chr12	5623294	5623472	388,12	downstream	204847	ENSMUSG00000037735	2810032G03Rik
chrX	120289919	120290177	88,90	downstream	33701	ENSMUSG00000079450	3110007F17Rik
chrX	53389837	53389991	149,75	inside	88	ENSMUSG00000031125	3830403N18Rik
chrX	53390255	53390511	55,65	inside	506	ENSMUSG00000031125	3830403N18Rik
chrX	53392520	53392794	199,92	inside	2771	ENSMUSG00000031125	3830403N18Rik
chr14	13106058	13106385	57,37	upstream	-10665	ENSMUSG00000033111	3830406C13Rik
chr18	6567265	6567730	128,41	upstream	-36362	ENSMUSG00000039540	4921524L21Rik
chr14	9449845	9450176	60,05	inside	48908	ENSMUSG00000021747	4930452B06Rik
chr8	19756749	19756935	103,80	downstream	27003	ENSMUSG00000074456	4930467E23Rik
chr14	36914013	36914318	51,47	downstream	5868	ENSMUSG00000049546	4930474N05Rik
chr2	145860959	145861315	59,93	upstream	-6271	ENSMUSG00000037143	4930529M08Rik
chr12	92424143	92424539	56,67	upstream	-1931	ENSMUSG00000061533	4930534B04Rik
chr13	48112919	48113416	61,10	inside	1058	ENSMUSG00000047324	4931429P17Rik
chr13	105939365	105939657	56,34	downstream	67163	ENSMUSG00000021718	4933425L06Rik
chrX	135692814	135693118	96,29	upstream	-20953	ENSMUSG00000042525	4933428M09Rik
chr11	25813958	25814257	54,59	upstream	-143388	ENSMUSG00000032985	5730522E02Rik
chr11	25655387	25655685	54,83	inside	15183	ENSMUSG00000032985	5730522E02Rik
chr11	25940833	25941146	51,26	inside	40767	ENSMUSG00000032985	5730522E02Rik
chr7	86789436	86789737	58,99	upstream	-15646	ENSMUSG00000046591	5730590G19Rik
chr8	34579546	34579860	54,67	downstream	40365	ENSMUSG00000048544	5930422O12Rik
chr13	62693362	62693869	103,78	downstream	15497	ENSMUSG00000072066	6720489N17Rik
chr4	141676149	141676620	61,30	upstream	-2123	ENSMUSG00000040606	9030409G11Rik
chr1	139818246	139818529	53,70	upstream	-6501	ENSMUSG00000051480	A130050O07Rik
chr19	31935394	31935682	52,46	upstream	-7857	ENSMUSG00000052595	A1cf
chr7	68162578	68162983	67,17	downstream	204234	ENSMUSG00000078690	A230006K03Rik
chr9	99500355	99500714	50,18	upstream	-12566	ENSMUSG00000037953	A4gnt
chr6	60342902	60343172	52,06	downstream	10790	ENSMUSG00000046764	A530053G22Rik
chr3	26327898	26328161	53,87	downstream	97826	ENSMUSG00000074664	A830092H15Rik
chr19	8070398	8070749	56,43	upstream	-29881	ENSMUSG00000067656	AB056442
chr16	8555992	8556267	50,81	upstream	-17003	ENSMUSG00000057880	Abat
chr11	9147431	9147750	54,54	inside	53977	ENSMUSG00000004668	Abca13
chr12	120130640	120130988	61,85	inside	32334	ENSMUSG00000072791	Abcb5
chr18	10699551	10699864	51,26	inside	7143	ENSMUSG00000002475	Abhd3
chr6	48847021	48847397	147,77	inside	1768	ENSMUSG00000029811	Abp1
chr15	41721201	41721517	50,57	upstream	-19935	ENSMUSG00000042895	Abra
chr6	53178682	53179027	50,84	upstream	-58607	ENSMUSG00000090982	AC069141.1
chr13	38769474	38769812	59,49	downstream	14741	ENSMUSG00000090804	AC069562.1
chr10	98935028	98935496	67,50	downstream	28664	ENSMUSG00000090665	AC101882.1
chr10	90861265	90861567	79,75	downstream	170334	ENSMUSG00000090412	AC122188.1
chr5	63370697	63370925	75,75	downstream	165635	ENSMUSG00000090326	AC122231.1
chr18	8736617	8736980	156,66	downstream	42542	ENSMUSG00000092097	AC122826.1
chr12	49337939	49338296	50,60	downstream	323019	ENSMUSG00000091277	AC122891.1
chr12	49069943	49070350	53,28	downstream	591015	ENSMUSG00000091277	AC122891.1
chr13	118047682	118048038	50,81	upstream	-37800	ENSMUSG00000091423	AC123935.1

chr7	130174919	130175278	52,40	downstream	21293	ENSMUSG00000090938	AC125221.1
chr14	121085375	121085723	50,17	upstream	-63199	ENSMUSG00000090284	AC125405.1
chr17	69797443	69797675	52,00	inside	8777	ENSMUSG00000091636	AC127693.1
chr17	63060701	63061027	67,48	upstream	-104645	ENSMUSG00000090425	AC134243.1
chrY	1642445	1642799	91,19	upstream	-212564	ENSMUSG00000091571	AC134524.1
chrY	1649649	1649902	90,78	upstream	-205360	ENSMUSG00000091571	AC134524.1
chrY	1655739	1655964	65,02	upstream	-199270	ENSMUSG00000091571	AC134524.1
chrY	1655984	1656328	142,98	upstream	-199025	ENSMUSG00000091571	AC134524.1
chrY	1670846	1671240	100,80	upstream	-184163	ENSMUSG00000091571	AC134524.1
chrY	1672383	1672639	95,83	upstream	-182626	ENSMUSG00000091571	AC134524.1
chrY	1714724	1715049	62,73	upstream	-140285	ENSMUSG00000091571	AC134524.1
chrY	1717189	1717450	94,88	upstream	-137820	ENSMUSG00000091571	AC134524.1
chrY	1743198	1743523	112,71	upstream	-111811	ENSMUSG00000091571	AC134524.1
chrY	1744908	1745369	97,16	upstream	-110101	ENSMUSG00000091571	AC134524.1
chrY	1751813	1752268	535,01	upstream	-103196	ENSMUSG00000091571	AC134524.1
chrY	1752554	1752717	342,77	upstream	-102455	ENSMUSG00000091571	AC134524.1
chrY	1755407	1755620	185,55	upstream	-99602	ENSMUSG00000091571	AC134524.1
chrY	1767073	1767380	95,24	upstream	-87936	ENSMUSG00000091571	AC134524.1
chrY	1769120	1769630	444,13	upstream	-85889	ENSMUSG00000091571	AC134524.1
chrY	1787891	1791342	3100,00	upstream	-67118	ENSMUSG00000091571	AC134524.1
chrY	1791414	1792187	773,86	upstream	-63595	ENSMUSG00000091571	AC134524.1
chrY	1792281	1793233	3188,19	upstream	-62728	ENSMUSG00000091571	AC134524.1
chrY	1793391	1793838	658,25	upstream	-61618	ENSMUSG00000091571	AC134524.1
chr8	103726983	103727236	51,46	upstream	-7452	ENSMUSG00000091838	AC142450.1
chr8	103776403	103776708	53,13	downstream	41968	ENSMUSG00000091838	AC142450.1
chr12	37301666	37301922	50,68	upstream	-192898	ENSMUSG00000091007	AC145348.1
chrY	2473480	2473766	200,43	upstream	-76782	ENSMUSG00000090600	AC149585.1
chrY	2474655	2475008	161,23	upstream	-75607	ENSMUSG00000090600	AC149585.1
chrY	2475579	2475956	181,52	upstream	-74683	ENSMUSG00000090600	AC149585.1
chrY	2476958	2477545	424,04	upstream	-73304	ENSMUSG00000090600	AC149585.1
chrY	2477850	2478167	292,48	upstream	-72412	ENSMUSG00000090600	AC149585.1
chrY	2479862	2480270	181,02	upstream	-70400	ENSMUSG00000090600	AC149585.1
chrY	2482531	2482968	152,80	upstream	-67731	ENSMUSG00000090600	AC149585.1
chrY	2486438	2486704	124,13	upstream	-63824	ENSMUSG00000090600	AC149585.1
chrY	2488712	2488977	175,35	upstream	-61550	ENSMUSG00000090600	AC149585.1
chrY	2495873	2496134	113,33	upstream	-54389	ENSMUSG00000090600	AC149585.1
chrY	2501463	2501707	270,67	upstream	-48799	ENSMUSG00000090600	AC149585.1
chrY	2503177	2503585	312,49	upstream	-47085	ENSMUSG00000090600	AC149585.1
chrY	2528844	2529206	286,39	upstream	-21418	ENSMUSG00000090600	AC149585.1
chrY	2551782	2552037	96,22	inside	1520	ENSMUSG00000090600	AC149585.1
chrY	2565450	2565957	148,73	downstream	15188	ENSMUSG00000090600	AC149585.1
chrY	2569417	2569634	73,56	downstream	19155	ENSMUSG00000090600	AC149585.1
chrY	2603489	2603818	77,04	downstream	53227	ENSMUSG00000090600	AC149585.1
chrY	2604347	2604802	577,69	downstream	54085	ENSMUSG00000090600	AC149585.1
chrY	2606773	2607120	93,39	downstream	56511	ENSMUSG00000090600	AC149585.1
chrY	2691309	2691456	91,92	downstream	141047	ENSMUSG00000090600	AC149585.1
chrY	2777498	2777805	596,67	downstream	227236	ENSMUSG00000090600	AC149585.1
chrY	2779573	2784690	3100,00	downstream	229311	ENSMUSG00000090600	AC149585.1
chrY	2785296	2786428	1027,50	downstream	235034	ENSMUSG00000090600	AC149585.1
chrY	2786914	2790112	1824,25	downstream	236652	ENSMUSG00000090600	AC149585.1
chrY	2790333	2792016	337,50	downstream	240071	ENSMUSG00000090600	AC149585.1
chrY	2792161	2792559	272,35	downstream	241899	ENSMUSG00000090600	AC149585.1
chrY	2792668	2793867	1019,60	downstream	242406	ENSMUSG00000090600	AC149585.1
chrY	2793896	2797434	3100,00	downstream	243634	ENSMUSG00000090600	AC149585.1
chrY	2847373	2847783	96,92	downstream	297111	ENSMUSG00000090600	AC149585.1
chrY	2848108	2862254	3100,00	downstream	297846	ENSMUSG00000090600	AC149585.1
chrY	2862336	2862804	53,52	downstream	312074	ENSMUSG00000090600	AC149585.1
chrY	2862959	2863459	764,37	downstream	312697	ENSMUSG00000090600	AC149585.1
chrY	2863604	2864047	274,80	downstream	313342	ENSMUSG00000090600	AC149585.1
chrY	2864249	2865339	373,64	downstream	313987	ENSMUSG00000090600	AC149585.1
chrY	2865365	2865826	404,88	downstream	315103	ENSMUSG00000090600	AC149585.1
chrY	2865936	2867303	2667,93	downstream	315674	ENSMUSG00000090600	AC149585.1
chrY	2867318	2867500	336,64	downstream	317056	ENSMUSG00000090600	AC149585.1
chrY	2867682	2868621	648,38	downstream	317420	ENSMUSG00000090600	AC149585.1
chrY	2868994	2869501	135,33	downstream	318732	ENSMUSG00000090600	AC149585.1
chrY	2869613	2871538	1289,01	downstream	319351	ENSMUSG00000090600	AC149585.1
chrY	2871594	2872445	2017,58	downstream	321332	ENSMUSG00000090600	AC149585.1

chrY	2872646	2873538	253,85	downstream	322384	ENSMUSG00000090600	AC149585.1
chrY	2875023	2884293	1233,11	downstream	324761	ENSMUSG00000090600	AC149585.1
chrY	2884340	2886225	3100,00	downstream	334078	ENSMUSG00000090600	AC149585.1
chrY	2886243	2894078	3100,00	downstream	335981	ENSMUSG00000090600	AC149585.1
chrY	2894114	2895551	2492,17	downstream	343852	ENSMUSG00000090600	AC149585.1
chrY	2895588	2896364	485,73	downstream	345326	ENSMUSG00000090600	AC149585.1
chrY	2896521	2896944	296,19	downstream	346259	ENSMUSG00000090600	AC149585.1
chrY	2897052	2901257	3100,00	downstream	346790	ENSMUSG00000090600	AC149585.1
chr18	78573061	78573413	51,67	downstream	26568	ENSMUSG00000090451	AC151578.1
chr6	146134984	146135335	59,94	upstream	-5525	ENSMUSG00000090911	AC153574.1
chr17	20537127	20537344	67,81	upstream	-19000	ENSMUSG00000091151	AC154379.1
chr16	59603839	59604140	55,47	upstream	-26147	ENSMUSG00000091562	AC154473.2
chr6	117333548	117333904	50,81	upstream	-169482	ENSMUSG00000090549	AC155646.1
chr7	94115842	94116146	63,22	upstream	-29133	ENSMUSG00000090362	AC158401.1
chr7	49473800	49474208	66,47	upstream	-41623	ENSMUSG00000090967	AC158569.1
chr14	124423005	124423277	51,56	downstream	18428	ENSMUSG00000090286	AC161001.1
chr7	13295902	13296215	54,91	inside	22552	ENSMUSG00000090762	AC161211.1
chr8	74352561	74352843	53,95	downstream	28547	ENSMUSG00000091391	AC164531.1
chr8	74337195	74337437	59,73	downstream	43913	ENSMUSG00000091391	AC164531.1
chr8	74336220	74336649	57,59	downstream	44888	ENSMUSG00000091391	AC164531.1
chr16	9007276	9007603	63,18	upstream	-10817	ENSMUSG00000090530	AC165274.1
chr16	75134837	75135137	54,34	downstream	439648	ENSMUSG00000090978	AC166995.1
chr5	114646636	114646917	53,57	upstream	-3152	ENSMUSG00000042010	Acacb
chr9	104024219	104024672	53,98	upstream	-1950	ENSMUSG00000090150	Acad11
chr4	49414258	49414592	280,70	inside	4357	ENSMUSG00000060317	Acnat2
chr7	126766070	126766414	58,06	inside	4743	ENSMUSG00000033533	Acsm1
chr15	100985169	100985581	55,59	downstream	17138	ENSMUSG00000000530	Acvrl1
chr8	58229330	58229625	55,58	downstream	156415	ENSMUSG00000046258	Adam29
chr14	69178524	69178988	153,88	downstream	21619	ENSMUSG00000022057	Adamdec1
chr15	11063915	11064233	64,55	downstream	69129	ENSMUSG00000047497	Adamts12
chr9	30693168	30693505	59,73	downstream	36869	ENSMUSG00000033453	Adamts15
chr9	30555945	30556309	53,54	downstream	174092	ENSMUSG00000033453	Adamts15
chr7	74009070	74009620	59,43	inside	14477	ENSMUSG00000058145	Adamts17
chr15	94297406	94297653	51,47	upstream	-1557	ENSMUSG00000022449	Adamts20
chr6	92842001	92842346	75,60	inside	9434	ENSMUSG00000030022	Adamts9
chr13	8696838	8697192	52,43	inside	138489	ENSMUSG00000052551	Adarb2
chr6	55506852	55507181	56,89	downstream	96622	ENSMUSG00000029778	Adcyap1r1
chr14	21925895	21926223	61,97	inside	54040	ENSMUSG00000039197	Adk
chr18	62254901	62255289	61,82	downstream	84712	ENSMUSG00000045730	Adrb2
chr5	113423385	113423681	50,53	inside	21149	ENSMUSG00000042249	Adrbk2
chr2	179896825	179897243	62,09	upstream	-9365	ENSMUSG00000039041	Adrm1
chr7	84300995	84301308	51,26	downstream	745047	ENSMUSG00000025754	Agbl1
chr17	12180922	12181315	50,85	upstream	-130648	ENSMUSG00000023827	Agpat4
chr6	29825628	29825904	55,49	inside	5339	ENSMUSG00000029772	Ahcyl2
chr10	20724365	20724735	70,96	upstream	-36261	ENSMUSG00000019986	Ahi1
chr10	13439186	13439495	56,21	inside	124906	ENSMUSG00000019806	Aig1
chr7	82658841	82659261	55,65	inside	58087	ENSMUSG00000066406	Akap13
chr12	53886205	53886761	70,98	upstream	-10618	ENSMUSG00000061603	Akap6
chr12	53925468	53925900	105,54	inside	28645	ENSMUSG00000061603	Akap6
chr13	4559069	4559325	55,75	upstream	-14252	ENSMUSG00000021207	Akr1c21
chr13	4560331	4560904	1075,78	upstream	-12990	ENSMUSG00000021207	Akr1c21
chr11	84070424	84070754	66,42	downstream	33697	ENSMUSG00000090870	AL596252.1
chr13	25816312	25816683	56,50	upstream	-42426	ENSMUSG00000090410	AL606965.1
chr16	52653153	52653660	99,96	upstream	-198966	ENSMUSG00000022636	Alcam
chr6	90442670	90443003	53,59	inside	6249	ENSMUSG00000030088	Aldh1l1
chr10	21069973	21070365	56,40	upstream	-27124	ENSMUSG00000037542	Aldh8a1
chr10	21075847	21076223	85,89	upstream	-21250	ENSMUSG00000037542	Aldh8a1
chr10	35461335	35461752	78,65	upstream	-29638	ENSMUSG00000063953	Amd2
chr15	97266947	97267365	78,40	upstream	-189229	ENSMUSG00000048218	Amigo2
chr10	68926726	68927013	52,70	upstream	-70244	ENSMUSG00000069601	Ank3
chr8	73946662	73947229	56,08	downstream	15072	ENSMUSG00000046295	Ankle1
chr6	118569836	118570206	52,25	upstream	-57562	ENSMUSG00000007827	Ankrd26
chr7	99772238	99772467	52,84	inside	12532	ENSMUSG00000041343	Ankrd42
chr15	95822422	95822796	51,42	downstream	48837	ENSMUSG00000064210	Ano6
chr8	64649562	64649925	122,89	upstream	-47647	ENSMUSG00000031635	Anxa10
chr1	58350269	58350687	57,47	inside	15099	ENSMUSG00000079554	Aox3l1
chr13	95196190	95196546	55,62	inside	67275	ENSMUSG00000021686	Ap3b1

chr14	27439406	27439696	64,89	upstream	-18154	ENSMUSG00000021877	Arf4
chr2	44075350	44075597	53,06	inside	46480	ENSMUSG00000049744	Arhgap15
chr7	130463679	130464065	55,38	inside	49750	ENSMUSG00000030766	Arhgap17
chr9	57669340	57669632	51,48	upstream	-906	ENSMUSG00000004661	Arid3b
chr1	90744935	90745236	56,21	upstream	-146139	ENSMUSG00000049866	Arl4c
chr18	7323049	7323235	215,95	upstream	-25150	ENSMUSG00000061802	Armc4
chr18	7322540	7322901	129,57	upstream	-24641	ENSMUSG00000061802	Armc4
chr18	7321279	7321572	815,01	upstream	-23380	ENSMUSG00000061802	Armc4
chr18	7320696	7321116	215,03	upstream	-22797	ENSMUSG00000061802	Armc4
chr18	7320092	7320447	269,98	upstream	-22193	ENSMUSG00000061802	Armc4
chr8	55627729	55628152	119,52	upstream	-7955	ENSMUSG00000031519	Asb5
chr1	53442998	53443310	76,68	upstream	-33402	ENSMUSG00000026095	Asnsd1
chr10	44044378	44044735	64,46	upstream	-21583	ENSMUSG00000038160	Atg5
chr17	80210787	80211056	52,32	downstream	84581	ENSMUSG00000059811	Atl2
chr9	105428919	105429265	50,61	inside	730	ENSMUSG00000032570	Atp2c1
chr2	114253214	114253540	57,62	downstream	118413	ENSMUSG00000057147	Atpbd4
chr14	78492457	78492713	50,68	upstream	-3643	ENSMUSG00000045655	AU021034
chr6	13655667	13655822	89,22	upstream	-27701	ENSMUSG00000042742	B630005N14Rik
chr9	59203165	59203595	75,40	upstream	-1850	ENSMUSG00000025235	Bbs4
chr9	22492230	22492526	60,29	inside	9036	ENSMUSG00000035919	Bbs9
chr17	16571753	16572105	60,88	upstream	-516750	ENSMUSG00000060149	BC002059
chr17	16612820	16613234	58,03	upstream	-475683	ENSMUSG00000060149	BC002059
chr17	16849663	16850116	138,24	upstream	-238840	ENSMUSG00000060149	BC002059
chr12	101394777	101395216	64,05	inside	3086	ENSMUSG00000021179	BC002230
chr4	126909818	126910232	58,17	upstream	-11210	ENSMUSG00000042380	BC003266
chr10	39704719	39705054	54,99	inside	1726	ENSMUSG00000038528	BC021785
chr15	57093778	57094090	56,22	inside	2914	ENSMUSG00000022366	BC026439
chr16	44717391	44717750	54,63	upstream	-7023	ENSMUSG00000036208	BC027231
chr8	67628921	67629212	51,73	inside	24301	ENSMUSG00000074300	BC030870
chr13	68304416	68304656	56,21	downstream	340142	ENSMUSG00000064063	BC048507
chr11	85194699	85195054	60,17	inside	26304	ENSMUSG00000059439	Bcas3
chr2	126908741	126909033	52,43	inside	2728	ENSMUSG00000001999	Blvra
chr9	75736734	75737068	55,71	downstream	60905	ENSMUSG00000032179	Bmp5
chr9	22799978	22800331	60,65	upstream	-227542	ENSMUSG00000031963	Bmper
chr2	27357949	27358372	135,30	inside	5233	ENSMUSG00000026918	Brd3
chr2	27355105	27357940	2086,76	inside	8077	ENSMUSG00000026918	Brd3
chr2	27354355	27355028	153,94	inside	8827	ENSMUSG00000026918	Brd3
chr2	27351129	27354040	699,30	inside	12053	ENSMUSG00000026918	Brd3
chr8	90953709	90954078	55,90	upstream	-67616	ENSMUSG00000031660	Brd7
chr19	37067365	37067765	53,89	inside	66796	ENSMUSG00000040565	Btaf1
chr5	107890427	107890724	55,08	inside	9252	ENSMUSG00000070632	Btbd8
chr16	45262726	45263003	50,32	downstream	38276	ENSMUSG00000052013	Btla
chrX	120146663	120146959	76,10	upstream	-3385	ENSMUSG00000092115	BX005170.1
chrX	120145051	120145322	51,81	upstream	-1773	ENSMUSG00000092115	BX005170.1
chrX	120143762	120144220	95,59	upstream	-484	ENSMUSG00000092115	BX005170.1
chrX	120137782	120138107	52,89	downstream	5496	ENSMUSG00000092115	BX005170.1
chrX	120133977	120134284	89,43	downstream	9301	ENSMUSG00000092115	BX005170.1
chrX	120129902	120130079	111,13	downstream	13376	ENSMUSG00000092115	BX005170.1
chrX	120128796	120129740	360,15	downstream	14482	ENSMUSG00000092115	BX005170.1
chrX	120128171	120128527	129,17	downstream	15107	ENSMUSG00000092115	BX005170.1
chrX	120126942	120128157	512,91	downstream	16336	ENSMUSG00000092115	BX005170.1
chrX	120126672	120126895	260,35	downstream	16606	ENSMUSG00000092115	BX005170.1
chrX	120126422	120126612	164,33	downstream	16856	ENSMUSG00000092115	BX005170.1
chrX	120125429	120126151	719,35	downstream	17849	ENSMUSG00000092115	BX005170.1
chrX	120125108	120125330	123,46	downstream	18170	ENSMUSG00000092115	BX005170.1
chrX	120122914	120123325	537,52	downstream	20364	ENSMUSG00000092115	BX005170.1
chrX	120120510	120121516	431,59	downstream	22768	ENSMUSG00000092115	BX005170.1
chrX	120118791	120119798	1082,55	downstream	24487	ENSMUSG00000092115	BX005170.1
chrX	120117543	120118691	744,92	downstream	25735	ENSMUSG00000092115	BX005170.1
chrX	120116930	120117483	977,32	downstream	26348	ENSMUSG00000092115	BX005170.1
chrX	120115722	120116819	2226,28	downstream	27556	ENSMUSG00000092115	BX005170.1
chrX	120114500	120114879	402,72	downstream	28778	ENSMUSG00000092115	BX005170.1
chrX	120113964	120114203	104,20	downstream	29314	ENSMUSG00000092115	BX005170.1
chrX	120113566	120113860	230,78	downstream	29712	ENSMUSG00000092115	BX005170.1
chrX	120112454	120113170	681,50	downstream	30824	ENSMUSG00000092115	BX005170.1
chrX	120347441	120347596	114,23	upstream	-11351	ENSMUSG00000091821	BX679668.1
chrX	120346947	120347266	84,56	upstream	-10857	ENSMUSG00000091821	BX679668.1

chrX	120340474	120340647	184,92	upstream	-4384	ENSMUSG00000091821	BX679668.1
chrX	120337805	120338067	367,93	upstream	-1715	ENSMUSG00000091821	BX679668.1
chrX	120334940	120335140	314,71	overlapEnd	1150	ENSMUSG00000091821	BX679668.1
chrX	120333264	120333574	77,30	downstream	2826	ENSMUSG00000091821	BX679668.1
chr5	3590300	3590565	53,35	downstream	1842	ENSMUSG00000040302	C030048B08Rik
chr15	10868766	10869105	67,81	upstream	-13321	ENSMUSG00000058914	C1qtnf3
chr6	124514082	124514385	51,47	upstream	-21705	ENSMUSG00000038521	C1s
chr1	49782131	49782526	74,43	downstream	480671	ENSMUSG00000051616	C230029F24Rik
chr2	149779451	149779781	52,00	upstream	-122502	ENSMUSG00000074737	C530025M09Rik
chr5	94109937	94110319	51,81	upstream	-9360	ENSMUSG00000070686	C87414
chr18	11985530	11985810	54,56	upstream	-12199	ENSMUSG00000040957	Cables1
chr4	100512332	100512706	51,42	inside	63052	ENSMUSG00000028532	Cachd1
chr9	47375650	47375908	50,17	inside	37194	ENSMUSG00000032076	Cadm1
chr16	68007372	68007717	67,50	upstream	-386219	ENSMUSG00000064115	Cadm2
chr18	61145548	61146034	62,85	downstream	22294	ENSMUSG00000024617	Camk2a
chr3	126236358	126236732	60,41	upstream	-62862	ENSMUSG00000053819	Camk2d
chr4	8173507	8173872	52,43	upstream	-7319	ENSMUSG00000041261	Car8
chr5	141596558	141596904	55,23	upstream	-120008	ENSMUSG00000036526	Card11
chr6	4546644	4547067	55,65	upstream	-4196	ENSMUSG00000015189	Casd1
chr6	83053075	83053797	55,27	inside	1464	ENSMUSG00000079511	Ccdc142
chr5	20918904	20919198	51,01	inside	11591	ENSMUSG00000064280	Ccdc146
chr1	54388930	54389218	57,37	inside	2094	ENSMUSG00000025983	Ccdc150
chr4	133714737	133715022	51,22	upstream	-1901	ENSMUSG00000037443	Ccdc21
chr11	102564747	102565065	59,59	upstream	-5651	ENSMUSG00000020925	Ccdc43
chr16	27409564	27409961	59,79	downstream	19510	ENSMUSG00000038127	Ccdc50
chr16	27611267	27611642	58,03	downstream	221213	ENSMUSG00000038127	Ccdc50
chr16	27782990	27783287	50,30	downstream	392936	ENSMUSG00000038127	Ccdc50
chr10	87695591	87695905	60,60	downstream	31646	ENSMUSG00000020056	Ccdc53
chr9	121388893	121389213	109,68	downstream	15864	ENSMUSG00000032532	Cck
chr11	81952730	81953017	51,71	downstream	24043	ENSMUSG00000009185	Ccl8
chr9	123996945	123997375	291,31	upstream	-19744	ENSMUSG00000049103	Ccr2
chr11	99044297	99044688	51,72	upstream	-27906	ENSMUSG00000037944	Ccr7
chr9	104055832	104056136	52,91	upstream	-26859	ENSMUSG00000079355	Ccrl1
chr8	3905730	3906066	55,25	downstream	21068	ENSMUSG00000065987	Cd209b
chr15	23257551	23257817	53,09	inside	153932	ENSMUSG00000040420	Cdh18
chr15	14139210	14139506	50,53	upstream	-1035816	ENSMUSG00000039385	Cdh6
chr1	111856112	111856324	52,41	upstream	-22792	ENSMUSG00000026312	Cdh7
chr4	155011627	155011920	57,41	inside	3350	ENSMUSG00000029062	Cdk11b
chr13	64627962	64628311	66,47	downstream	94101	ENSMUSG00000021483	Cdk20
chr5	3363530	3363835	71,34	inside	19218	ENSMUSG00000040274	Cdk6
chr5	86463732	86464033	54,10	inside	30876	ENSMUSG00000029253	Cenpc1
chr1	157812223	157812472	68,59	inside	7794	ENSMUSG00000033671	Cep350
chr8	95570889	95571228	50,01	inside	1202	ENSMUSG00000071047	Ces1a
chr8	96122539	96122875	50,67	upstream	-62932	ENSMUSG00000058019	Ces5a
chr17	15856209	15856452	54,15	inside	14278	ENSMUSG00000023852	Chd1
chr8	93465630	93465910	64,69	upstream	-13317	ENSMUSG00000056608	Chd9
chr3	106059947	106060705	54,69	upstream	-37522	ENSMUSG00000040809	Chi3l3
chr3	105998399	105998842	52,59	downstream	15250	ENSMUSG00000063779	Chi3l4
chr5	75429115	75429422	56,21	inside	11684	ENSMUSG00000029229	Chic2
chr1	177601958	177602362	77,05	downstream	16526	ENSMUSG00000078185	Chml
chr1	38971361	38971639	50,08	upstream	-16356	ENSMUSG00000026080	Chst10
chr18	15875209	15875617	71,19	inside	1346	ENSMUSG00000047161	Chst9
chr8	63405956	63406173	50,97	upstream	-4199	ENSMUSG00000004319	Clcn3
chr17	72293190	72293507	64,29	downstream	161903	ENSMUSG00000024059	Clip4
chr5	39253525	39253824	56,21	inside	14407	ENSMUSG00000039315	Clnk
chr12	27267792	27268231	187,92	downstream	113723	ENSMUSG00000020638	Cmpk2
chr12	27298997	27299260	63,21	downstream	144928	ENSMUSG00000020638	Cmpk2
chr4	19040514	19040791	55,23	inside	9159	ENSMUSG00000073991	Cnbd1
chr6	102320904	102321194	56,85	inside	93757	ENSMUSG00000030075	Cntn3
chr6	46966492	46966798	57,71	upstream	-28567	ENSMUSG00000039419	Cntnap2
chr8	115456066	115456393	62,22	downstream	158512	ENSMUSG00000031772	Cntnap4
chr9	79096673	79097031	56,82	downstream	364104	ENSMUSG00000032332	Col12a1
chr1	82576593	82577010	54,24	inside	6774	ENSMUSG00000067158	Col4a4
chr1	82493655	82493885	52,56	downstream	89712	ENSMUSG00000067158	Col4a4
chr11	22845636	22846003	56,82	inside	11311	ENSMUSG00000051355	Commd1
chr1	88495733	88496061	57,72	inside	2418	ENSMUSG00000026240	Cops7b
chr11	63878943	63879475	54,93	inside	14025	ENSMUSG00000042148	Cox10

chr9	79590136	79590463	52,68	downstream	17524	ENSMUSG00000032330	Cox7a2
chr13	54485408	54486055	55,91	downstream	12695	ENSMUSG00000025867	Cplx2
chrX	120812236	120812422	58,53	downstream	6170	ENSMUSG00000091916	CR628367.1
chr10	94812892	94813451	53,90	upstream	-26125	ENSMUSG00000045867	Cradd
chr1	141189778	141190055	50,32	inside	35047	ENSMUSG00000063681	Crb1
chr1	65107921	65108233	61,12	downstream	1954	ENSMUSG00000067299	Crygd
chr8	15959644	15959946	51,47	inside	25168	ENSMUSG00000060924	Csmd1
chr2	149251892	149252285	60,68	downstream	20823	ENSMUSG00000033156	Cst10
chr19	31135725	31136183	71,96	upstream	-21606	ENSMUSG00000053536	Cstf2t
chr7	140205254	140205653	69,75	inside	1677	ENSMUSG00000030970	Ctbp2
chr15	30165264	30165670	71,68	inside	62916	ENSMUSG00000022240	Ctnnd2
chr13	60928187	60928520	60,72	upstream	-23982	ENSMUSG00000056728	Ctsll3
chr9	53437489	53437856	53,89	upstream	-7350	ENSMUSG00000032030	Cul5
chr5	91237876	91238209	55,95	downstream	14316	ENSMUSG00000029375	Cxcl15
chr7	27645043	27645469	74,93	downstream	19080	ENSMUSG00000005547	Cyp2a5
chr15	82535751	82536062	51,72	upstream	-14822	ENSMUSG00000075517	Cyp2d37-ps
chr5	4087208	4087650	69,29	downstream	16173	ENSMUSG00000001467	Cyp51
chr1	99573351	99573687	55,25	upstream	-14700	ENSMUSG00000044768	D1Ertd622e
chr3	42357106	42357502	51,23	downstream	810459	ENSMUSG00000025766	D3Ertd751e
chr17	14357108	14357457	56,66	upstream	-16270	ENSMUSG00000048826	Dact2
chr3	137640439	137640879	62,29	inside	4055	ENSMUSG00000028159	Dapp1
chr13	25195850	25196208	79,28	inside	7634	ENSMUSG00000035910	Dcdc2a
chr5	89212434	89212744	56,71	downstream	18413	ENSMUSG00000029366	Dck
chr2	3375916	3376228	51,47	downstream	5695	ENSMUSG00000026648	Dclre1c
chr6	119072927	119073208	54,20	upstream	-52343	ENSMUSG00000041477	Dcp1b
chr14	118268961	118269232	56,81	downstream	182505	ENSMUSG00000022129	Dct
chrX	140471924	140472305	58,82	upstream	-104070	ENSMUSG00000031285	Dcx
chrX	140469301	140469609	58,40	upstream	-101447	ENSMUSG00000031285	Dcx
chrX	140456302	140456670	156,85	upstream	-88448	ENSMUSG00000031285	Dcx
chrX	140455441	140455812	149,64	upstream	-87587	ENSMUSG00000031285	Dcx
chr4	141268830	141269100	55,61	inside	10504	ENSMUSG00000078515	Ddi2
chr2	130475608	130475832	59,28	downstream	4905	ENSMUSG00000068290	Ddrgk1
chr3	159022271	159022582	56,21	upstream	-136126	ENSMUSG00000028175	Depdc1a
chr14	79027406	79027681	60,86	inside	1675	ENSMUSG00000034731	Dgkh
chr9	110001762	110002096	53,37	inside	1829	ENSMUSG00000032480	Dhx30
chr11	101583012	101583456	70,41	upstream	-11221	ENSMUSG00000034931	Dhx8
chr7	98410559	98410806	58,27	upstream	-884	ENSMUSG00000052572	Dlg2
chrX	81689192	81689458	63,42	upstream	-155348	ENSMUSG00000045103	Dmd
chr14	27604519	27604784	53,35	downstream	20304	ENSMUSG00000021879	Dnahc12
chr18	90100051	90100348	50,30	upstream	-161131	ENSMUSG00000073514	Dok6
chr1	126127176	126127450	51,06	upstream	-185040	ENSMUSG00000036815	Dpp10
chr1	166751984	166752277	50,26	inside	25121	ENSMUSG00000026574	Dpt
chr16	97409190	97409541	56,43	upstream	-16831	ENSMUSG00000050272	Dscam
chr18	20356615	20356934	59,34	upstream	-49226	ENSMUSG00000034774	Dsg1c
chr8	31375968	31376239	72,81	upstream	-823975	ENSMUSG00000039661	Dusp26
chr8	31459194	31459445	51,99	upstream	-740749	ENSMUSG00000039661	Dusp26
chr8	32024238	32024614	59,95	upstream	-175705	ENSMUSG00000039661	Dusp26
chr6	5629669	5629990	54,07	upstream	-45970	ENSMUSG00000029757	Dync1i1
chr17	85038603	85038901	51,47	inside	12767	ENSMUSG00000024253	Dync2li1
chr4	49065783	49066044	54,40	upstream	-6362	ENSMUSG00000063446	E130309F12Rik
chr13	89317403	89317717	51,03	inside	356156	ENSMUSG00000034488	Edil3
chr13	42450251	42450585	52,43	downstream	53612	ENSMUSG00000021367	Edn1
chr13	42483371	42483606	50,28	downstream	86732	ENSMUSG00000021367	Edn1
chr13	42536675	42536972	55,08	downstream	140036	ENSMUSG00000021367	Edn1
chr17	63216270	63216547	55,23	inside	14223	ENSMUSG00000048915	Efna5
chr3	29058732	29059089	56,82	inside	77039	ENSMUSG00000063600	Egfem1
chr6	99627959	99628281	51,20	upstream	-11168	ENSMUSG00000030068	Eif4e3
chr5	135111910	135112218	50,26	inside	3289	ENSMUSG00000040731	Eif4h
chr11	64742763	64743049	52,95	upstream	-49777	ENSMUSG00000020549	Elac2
chr9	53853179	53853591	128,49	upstream	-30071	ENSMUSG00000041986	Elmod1
chr13	41299312	41299635	53,60	inside	16219	ENSMUSG00000021364	Elovl2
chr17	83723310	83723753	55,65	upstream	-26961	ENSMUSG00000032624	Eml4
chr3	129040172	129040470	54,83	upstream	-4534	ENSMUSG00000028024	Enpep
chr10	25188088	25188562	88,68	inside	26695	ENSMUSG00000019978	Epb4.1l2
chr17	69378229	69378527	59,76	upstream	-127921	ENSMUSG00000024044	Epb4.1l3
chr17	69514050	69514392	58,53	inside	7900	ENSMUSG00000024044	Epb4.1l3
chr16	64043407	64043906	97,06	upstream	-179423	ENSMUSG00000052504	Epha3

chr16	63806287	63806681	65,07	inside	57697	ENSMUSG00000052504	Epha3
chr5	84879549	84879899	61,37	upstream	-33142	ENSMUSG00000029245	Epha5
chr16	60090901	60091230	56,89	upstream	-85354	ENSMUSG00000055540	Epha6
chr17	32350836	32351162	69,96	upstream	-24342	ENSMUSG00000037577	Ephx3
chr4	109011489	109011833	58,06	upstream	-4189	ENSMUSG00000028552	Eps15
chr14	28404934	28405200	53,09	upstream	-30680	ENSMUSG00000040640	Erc2
chr18	32364735	32364995	51,47	upstream	-35219	ENSMUSG00000024382	Ercc3
chr14	33362907	33363210	51,47	inside	36200	ENSMUSG00000054051	Ercc6
chr8	14062630	14062942	51,49	inside	27671	ENSMUSG00000051978	Erich1
chr1	189591917	189592245	61,97	inside	158342	ENSMUSG00000026610	Esrrg
chr1	189594153	189594461	51,47	inside	160578	ENSMUSG00000026610	Esrrg
chr6	143151592	143151928	59,98	inside	35842	ENSMUSG00000030275	Etnk1
chr5	37802715	37803154	57,14	upstream	-4383	ENSMUSG00000050248	Evc2
chr5	108232457	108232800	73,09	upstream	-3892	ENSMUSG00000011831	Evi5
chr19	21778935	21779273	64,35	downstream	51136	ENSMUSG00000047368	Fam108b
chr13	78053529	78053850	63,77	downstream	205539	ENSMUSG00000064138	Fam172a
chr2	12297804	12298178	56,82	upstream	-19266	ENSMUSG00000026767	Fam188a
chr16	10433640	10434021	50,69	upstream	-4286	ENSMUSG00000050908	Fam18a
chr6	61736667	61737050	52,43	inside	216003	ENSMUSG00000039578	Fam190a
chr3	79720891	79721268	55,19	inside	31039	ENSMUSG00000027955	Fam198b
chr18	63277203	63277572	52,92	inside	39812	ENSMUSG00000041482	Fam38b
chr18	63461420	63461801	63,44	inside	84691	ENSMUSG00000041482	Fam38b
chr6	148899357	148899626	57,35	upstream	-4368	ENSMUSG00000039985	Fam60a
chr5	108389729	108390111	67,95	inside	12288	ENSMUSG00000029270	Fam69a
chr9	69993251	69993580	59,92	upstream	-2884	ENSMUSG00000032224	Fam81a
chr1	95416629	95416906	55,86	inside	7940	ENSMUSG00000034066	Farp2
chr9	16333055	16333349	60,82	upstream	-150374	ENSMUSG00000074505	Fat3
chr1	172922075	172922445	75,98	upstream	-15397	ENSMUSG00000026656	Fcgr2b
chr9	51756691	51757046	51,03	inside	14960	ENSMUSG00000032051	Fdx1
chr17	64367568	64367928	52,43	inside	80247	ENSMUSG00000000127	Fer
chr12	34464553	34464892	59,25	upstream	-148737	ENSMUSG00000046518	Ferd3l
chr14	124948344	124948680	59,98	inside	128005	ENSMUSG00000025551	Fgf14
chr8	41326886	41327260	66,00	downstream	45421	ENSMUSG00000031603	Fgf20
chr14	73031346	73031644	54,83	inside	20537	ENSMUSG00000033487	Fndc3a
chr8	83181608	83181974	57,53	inside	46629	ENSMUSG00000042353	Frem3
chr16	37770191	37770596	62,55	upstream	-6768	ENSMUSG00000022816	Fstl1
chr15	6590425	6590839	60,56	upstream	-3261	ENSMUSG00000022148	Fyb
chr10	39201658	39202125	71,71	inside	26937	ENSMUSG00000019843	Fyn
chr5	4740618	4740946	57,13	downstream	17598	ENSMUSG00000044674	Fzd1
chr5	4505191	4505476	68,55	downstream	253025	ENSMUSG00000044674	Fzd1
chr16	84871972	84872328	60,15	downstream	11682	ENSMUSG00000008976	Gabpa
chr7	64799306	64799613	61,10	upstream	-33927	ENSMUSG00000055078	Gabra5
chr16	42229065	42229388	53,60	downstream	47120	ENSMUSG00000047261	Gap43
chr13	60259545	60259855	50,94	downstream	19083	ENSMUSG00000052957	Gas1
chr10	57744406	57744645	50,10	upstream	-4269	ENSMUSG00000038039	Gcc2
chr15	7741528	7741855	62,22	upstream	-19483	ENSMUSG00000022144	Gdnf
chr6	87007653	87008086	71,15	inside	14768	ENSMUSG00000029992	Gfpt1
chr3	96888403	96888876	80,52	downstream	52064	ENSMUSG00000057123	Gja5
chr10	5232380	5232612	57,30	downstream	44968	ENSMUSG00000061261	Gm10097
chr14	88357262	88357581	54,54	downstream	166353	ENSMUSG00000062611	Gm10119
chr9	96804184	96804481	50,30	downstream	8151	ENSMUSG00000062933	Gm10123
chr4	96958547	96958994	71,36	upstream	-108690	ENSMUSG00000066990	Gm10192
chr5	29057848	29058190	58,53	downstream	192788	ENSMUSG00000070408	Gm10290
chr8	77927595	77927866	51,81	downstream	2870	ENSMUSG00000072432	Gm10358
chr5	109341614	109341885	84,25	downstream	47762	ENSMUSG00000072766	Gm10417
chr5	26421566	26422367	82,70	upstream	-5735	ENSMUSG00000073116	Gm10471
chr12	20471164	20471484	59,09	downstream	49365	ENSMUSG00000073184	Gm10479
chr5	10206739	10207054	60,35	upstream	-30090	ENSMUSG00000073226	Gm10482
chr7	90694320	90694593	56,28	downstream	3818	ENSMUSG00000074038	Gm10610
chr2	6834181	6834526	53,20	downstream	21946	ENSMUSG00000075538	Gm10855
chr7	73509365	73509771	71,15	upstream	-1426	ENSMUSG00000078677	Gm10974
chr13	102119328	102119752	65,29	upstream	-333597	ENSMUSG00000078939	Gm11016
chr12	114640448	114640693	53,60	upstream	-14647	ENSMUSG00000078993	Gm11029
chr12	75847206	75847527	58,84	upstream	-429825	ENSMUSG00000079061	Gm11042
chr9	85097796	85098073	50,32	upstream	-108584	ENSMUSG00000079433	Gm11114
chr1	27089347	27089719	51,83	upstream	-9999	ENSMUSG00000079640	Gm11161
chr13	26560475	26560773	70,10	upstream	-23943	ENSMUSG00000060198	Gm11353

chr4	145702580	145703153	63,12	upstream	-6568	ENSMUSG00000056300	Gm13247
chr4	145680376	145681108	54,65	inside	2743	ENSMUSG00000056300	Gm13247
chr14	46934066	46934391	53,14	upstream	-65023	ENSMUSG00000079261	Gm15217
chr3	28780282	28780569	438,37	upstream	-11257	ENSMUSG00000074655	Gm1527
chr3	28781431	28782004	104,49	upstream	-10108	ENSMUSG00000074655	Gm1527
chr3	28782196	28782478	64,12	upstream	-9343	ENSMUSG00000074655	Gm1527
chr8	19960701	19961105	92,41	upstream	-1913	ENSMUSG00000074449	Gm15319
chr8	19939308	19940486	79,49	inside	19480	ENSMUSG00000074449	Gm15319
chr8	19937351	19937766	50,88	inside	21437	ENSMUSG00000074449	Gm15319
chr8	19935636	19936229	67,65	inside	23152	ENSMUSG00000074449	Gm15319
chr8	19929521	19930319	80,96	downstream	29267	ENSMUSG00000074449	Gm15319
chr7	46517513	46518299	80,67	inside	2391	ENSMUSG00000074169	Gm16387
chr17	76866405	76866772	52,89	upstream	-182204	ENSMUSG00000066958	Gm16391
chr18	42630857	42631134	65,55	downstream	30184	ENSMUSG00000056849	Gm16415
chr4	146915194	146915608	79,42	inside	867	ENSMUSG00000078160	Gm16503
chr8	5666994	5667248	51,47	downstream	27410	ENSMUSG00000043192	Gm1840
chr1	52122300	52122591	61,63	downstream	25610	ENSMUSG00000078291	Gm3940
chr14	114600431	114600673	108,06	upstream	-710549	ENSMUSG00000086415	Gm4487
chr9	57218866	57219289	63,07	downstream	36946	ENSMUSG00000066626	Gm5121
chr18	14432642	14432895	51,46	upstream	-149517	ENSMUSG00000055795	Gm5160
chr10	53206923	53207254	50,74	downstream	12153	ENSMUSG00000056219	Gm5423
chr13	4839050	4839303	56,58	downstream	68155	ENSMUSG00000053499	Gm5444
chr6	40070144	40070456	55,74	inside	9892	ENSMUSG00000057716	Gm5567
chr7	46696568	46696889	69,53	downstream	4976	ENSMUSG00000046372	Gm5590
chr1	28793711	28794025	55,74	downstream	43386	ENSMUSG00000048411	Gm597
chr19	61262136	61262632	77,56	downstream	2343	ENSMUSG00000069475	Gm6020
chr16	27005229	27005521	51,48	upstream	-15169	ENSMUSG00000068428	Gm606
chr16	26901524	26901879	60,17	downstream	66807	ENSMUSG00000068428	Gm606
chr16	44212713	44213015	55,04	upstream	-5514	ENSMUSG00000068284	Gm608
chr14	111415257	111415528	51,81	downstream	45247	ENSMUSG00000060796	Gm6280
chr13	117012204	117012540	50,67	downstream	68231	ENSMUSG00000061669	Gm6404
chr18	14666126	14666453	52,68	upstream	-62496	ENSMUSG00000053740	Gm6457
chr19	23681823	23682136	60,86	upstream	-12409	ENSMUSG00000044424	Gm6472
chr15	59789047	59789436	66,26	downstream	37336	ENSMUSG00000071750	Gm7713
chr15	59724351	59724715	59,67	downstream	102032	ENSMUSG00000071750	Gm7713
chr14	45290711	45291002	247,49	upstream	-13679	ENSMUSG00000079265	Gm8257
chr14	45289826	45290183	194,88	upstream	-12794	ENSMUSG00000079265	Gm8257
chr3	60641388	60641697	82,15	upstream	-39546	ENSMUSG00000027694	Gm8325
chr17	9735097	9735414	50,34	downstream	120172	ENSMUSG00000072968	Gm8719
chr13	109565212	109565639	81,02	downstream	104439	ENSMUSG00000059751	Gm9000
chr12	19718962	19719387	65,40	downstream	106178	ENSMUSG00000078375	Gm9257
chr12	27621552	27621961	66,24	downstream	223759	ENSMUSG00000052076	Gm9866
chr9	60704762	60705089	54,49	upstream	-18755	ENSMUSG00000052143	Gm9869
chr9	60692730	60693071	58,77	upstream	-6723	ENSMUSG00000052143	Gm9869
chr1	30780726	30780878	159,38	upstream	-3920	ENSMUSG00000053185	Gm9898
chr2	125325827	125326162	55,48	upstream	-4998	ENSMUSG00000053615	Gm9913
chr1	42455637	42456146	59,67	downstream	169066	ENSMUSG00000053640	Gm9915
chr18	67187002	67187267	58,44	upstream	-60988	ENSMUSG00000024524	Gnal
chr18	67234541	67234791	82,15	upstream	-13449	ENSMUSG00000024524	Gnal
chr18	67235191	67235590	54,06	upstream	-12799	ENSMUSG00000024524	Gnal
chr8	96263215	96263558	53,65	upstream	-70651	ENSMUSG00000031748	Gnao1
chr13	13847175	13847531	61,35	upstream	-29151	ENSMUSG00000021303	Gng4
chr13	112202350	112202732	63,20	downstream	41240	ENSMUSG00000032745	Gpbp1
chr14	116074682	116074949	68,52	downstream	305929	ENSMUSG00000022112	Gpc5
chr12	76475489	76475801	56,21	downstream	42279	ENSMUSG00000048982	Gphb5
chr8	56200626	56200920	56,85	downstream	160545	ENSMUSG00000031517	Gpm6a
chr7	126288281	126288586	66,90	inside	39607	ENSMUSG00000066197	Gpr139
chr3	115962330	115962557	51,44	upstream	-5928	ENSMUSG00000068696	Gpr88
chr11	114734393	114734982	66,04	downstream	8587	ENSMUSG00000051043	Gprc5c
chr6	63914321	63914599	51,47	upstream	-112465	ENSMUSG00000071424	Grid2
chr6	63192030	63192448	55,33	upstream	-13840	ENSMUSG00000071424	Grid2
chr10	49117032	49117445	69,99	upstream	-154190	ENSMUSG00000056073	Grik2
chr10	49312711	49313028	50,34	upstream	-14602	ENSMUSG00000056073	Grik2
chr16	9758586	9758914	57,13	inside	234040	ENSMUSG00000059003	Grin2a
chr6	136081523	136081855	51,55	inside	40217	ENSMUSG00000030209	Grin2b
chr10	10551929	10552286	59,70	upstream	-83323	ENSMUSG00000019828	Grm1
chr10	10500084	10500387	53,61	upstream	-31478	ENSMUSG00000019828	Grm1

chr18	42144686	42145034	57,12	downstream	14017	ENSMUSG00000073574	Grxcr2
chr6	55923184	55923519	61,35	upstream	-44325	ENSMUSG00000002930	Gsbs
chr4	119314031	119314437	86,62	downstream	3722	ENSMUSG00000023247	Guca2a
chr9	3947942	3948357	141,54	downstream	189025	ENSMUSG00000041624	Gucy1a2
chr3	137484834	137485234	68,36	upstream	-42617	ENSMUSG00000037894	H2afz
chr17	36621376	36621971	997,84	upstream	-22097	ENSMUSG00000048231	H2-M10.4
chr10	22010939	22011207	57,62	downstream	17787	ENSMUSG00000075297	H60b
chr17	84194385	84194800	57,06	downstream	51745	ENSMUSG00000000673	Haao
chr10	45390205	45390557	56,82	upstream	-1115	ENSMUSG00000038822	Hace1
chr2	134265928	134266225	50,30	downstream	114115	ENSMUSG00000027261	Hao1
chr9	76149749	76150024	50,81	inside	21636	ENSMUSG00000032360	Hcrtr2
chr1	93900305	93900712	74,37	inside	3127	ENSMUSG00000026313	Hdac4
chr12	34903407	34903908	76,45	inside	155578	ENSMUSG00000004698	Hdac9
chr1	95353421	95353764	53,65	upstream	-11158	ENSMUSG00000034088	Hdlbp
chr12	53085516	53086072	68,39	upstream	-13208	ENSMUSG00000035181	Heatr5a
chr10	18332849	18333207	64,21	upstream	-66967	ENSMUSG00000019853	Hebp2
chr11	107531432	107531985	52,59	overlapStart	-496	ENSMUSG00000020721	Helz
chr5	16094040	16094365	52,43	inside	34672	ENSMUSG00000028864	Hgf
chr6	52502536	52502852	60,09	inside	4373	ENSMUSG00000029776	Hibadh
chr4	119601375	119601566	64,96	inside	114042	ENSMUSG00000028634	Hivep3
chr10	61844696	61845067	61,10	upstream	-2040	ENSMUSG00000037012	Hk1
chr10	119976381	119976698	53,95	upstream	-62856	ENSMUSG00000056758	Hmga2
chr9	83140906	83141228	58,59	upstream	-100614	ENSMUSG00000066456	Hmgn3
chr9	82943134	82943464	52,00	downstream	61298	ENSMUSG00000066456	Hmgn3
chr12	17777497	17777747	51,32	inside	79877	ENSMUSG00000071379	Hpcal1
chr18	13154019	13154406	76,50	upstream	-11480	ENSMUSG00000037346	Hrh4
chr18	13198855	13199020	50,19	downstream	33309	ENSMUSG00000037346	Hrh4
chr1	36196266	36196712	72,06	downstream	71021	ENSMUSG00000045216	Hs6st1
chr5	104379465	104379829	79,03	downstream	26880	ENSMUSG00000034528	Hsd17b13
chr18	50334672	50335036	58,08	upstream	-16306	ENSMUSG00000024507	Hsd17b4
chr3	98542533	98542859	52,91	upstream	-14034	ENSMUSG00000063730	Hsd3b2
chr13	106226332	106226651	59,34	upstream	-7441	ENSMUSG00000021721	Htr1a
chr13	106294264	106294601	59,73	downstream	60491	ENSMUSG00000021721	Htr1a
chr8	25610197	25610530	65,08	downstream	41071	ENSMUSG00000031549	Ido2
chr3	151375664	151376034	66,00	downstream	37182	ENSMUSG00000028037	Ifi44
chr2	62471081	62471393	52,43	inside	13187	ENSMUSG00000026896	Ifih1
chr6	138816805	138817017	63,58	upstream	-209840	ENSMUSG00000046717	Igbp1b
chr7	75322290	75322697	50,12	inside	28833	ENSMUSG00000005533	Igf1r
chr6	67266043	67266287	59,14	inside	23645	ENSMUSG00000018341	Il12rb2
chr1	40599624	40599970	53,45	inside	1087	ENSMUSG00000026068	Il18rap
chr16	26687626	26687959	55,95	inside	105832	ENSMUSG00000022514	Il1rap
chr7	29279073	29279441	335,15	downstream	16401	ENSMUSG00000059128	Il28a
chr3	7666447	7666762	50,80	upstream	-52725	ENSMUSG00000040329	Il7
chr4	98178649	98178947	50,07	upstream	-7013	ENSMUSG00000061859	Inadl
chr8	83962762	83963016	51,20	inside	96301	ENSMUSG00000037940	Inpp4b
chr13	107637829	107638117	57,37	upstream	-482	ENSMUSG00000078933	Ipo11
chr1	137372048	137372359	51,71	upstream	-44972	ENSMUSG00000041879	Ipo9
chr3	67561659	67561997	58,35	upstream	-134483	ENSMUSG00000051777	Iqcj
chr6	90610379	90610748	52,07	downstream	7614	ENSMUSG00000034312	Iqsec1
chr9	54682610	54682988	54,97	upstream	-28952	ENSMUSG00000032293	Ireb2
chr8	47903491	47903930	82,47	inside	78392	ENSMUSG00000031627	Irf2
chr13	30912463	30912784	63,77	downstream	71368	ENSMUSG00000021356	Irf4
chr2	12248862	12249193	54,08	upstream	-25307	ENSMUSG00000026768	Itga8
chr13	44834063	44834309	53,33	inside	7791	ENSMUSG00000038518	Jarid2
chr16	34217764	34218057	61,10	downstream	34637	ENSMUSG00000061751	Kalrn
chr6	133851917	133852176	54,93	upstream	-48232	ENSMUSG00000032758	Kap
chr3	106975644	106975963	64,29	upstream	-10330	ENSMUSG00000042861	Kcna10
chr17	83973004	83973362	64,21	downstream	58231	ENSMUSG00000045053	Kcng3
chr12	76188395	76188795	68,36	inside	89924	ENSMUSG00000034402	Kcnh5
chr5	49765087	49765418	61,21	upstream	-88508	ENSMUSG00000029088	Kcnip4
chr18	45756263	45756601	59,49	inside	36455	ENSMUSG00000054477	Kcnn2
chr1	22014203	22014546	57,16	upstream	-62180	ENSMUSG00000028033	Kcnq5
chr15	34743097	34743355	50,17	upstream	-24013	ENSMUSG00000050963	Kcns2
chr18	15400620	15400995	61,35	upstream	-90665	ENSMUSG00000036225	Kctd1
chr1	190918178	190918476	50,07	upstream	-86459	ENSMUSG00000026608	Kctd3
chr6	41678197	41678489	51,48	upstream	-23859	ENSMUSG00000029866	Kel
chr2	49497172	49497430	55,20	inside	22338	ENSMUSG00000026764	Kif5c

chr17	49785720	49786099	80,73	inside	31179	ENSMUSG00000023999	Kif6
chr15	38210965	38211360	50,21	downstream	18501	ENSMUSG00000037465	Klf10
chr7	51435860	51436254	60,46	upstream	-7746	ENSMUSG00000063713	Klk1b24
chr6	129882879	129883297	71,15	upstream	-15211	ENSMUSG00000030173	Klra5
chr17	88918133	88918474	53,02	upstream	-11331	ENSMUSG00000034709	Klraq1
chr16	89525113	89525367	51,20	upstream	-16545	ENSMUSG00000056706	Krtap7-1
chr14	48249004	48249258	56,82	upstream	-34427	ENSMUSG00000021843	Ktn1
chr10	27332794	27333123	66,68	inside	3954	ENSMUSG00000019899	Lama2
chr8	75935736	75936072	54,16	upstream	-58297	ENSMUSG00000004383	Large
chr9	123323261	123323680	78,14	inside	47203	ENSMUSG00000035202	Lars2
chr3	92419974	92420437	161,23	downstream	5370	ENSMUSG00000086848	Lce6a
chr14	80026901	80027259	79,28	downstream	15101	ENSMUSG00000022025	Lect1
chr3	52894900	52895301	53,83	inside	49395	ENSMUSG00000048332	Lhfp
chr1	140694904	140695196	51,48	downstream	44102	ENSMUSG00000019230	Lhx9
chr10	51254533	51254860	52,68	downstream	43709	ENSMUSG00000062593	Lilrb4
chr10	57779864	57780116	51,72	upstream	-6350	ENSMUSG00000019920	Lims1
chr10	45142075	45142538	67,76	inside	47932	ENSMUSG00000063804	Lin28b
chr4	36196126	36196548	55,65	upstream	-112630	ENSMUSG00000045083	Lingo2
chr4	35803274	35803578	53,37	upstream	-10829	ENSMUSG00000045083	Lingo2
chr4	35764053	35764370	86,16	inside	28392	ENSMUSG00000045083	Lingo2
chr1	24864519	24864997	184,59	downstream	179136	ENSMUSG00000073725	Lmbrd1
chr3	145883558	145883862	51,24	upstream	-367	ENSMUSG00000036832	Lpar3
chr1	193548279	193548576	51,47	inside	5879	ENSMUSG00000026623	Lpgat1
chr3	148630856	148631110	56,16	upstream	-13257	ENSMUSG00000028184	Lphn2
chr16	24651737	24652045	62,16	upstream	-70204	ENSMUSG00000033306	Lpp
chr16	24739121	24739451	52,00	inside	17180	ENSMUSG00000033306	Lpp
chr10	125550199	125550473	51,06	downstream	146924	ENSMUSG00000020105	Lrig3
chr10	125633333	125633679	62,34	downstream	230058	ENSMUSG00000020105	Lrig3
chr10	125817400	125817774	59,20	downstream	414125	ENSMUSG00000020105	Lrig3
chr6	124733906	124734333	50,24	upstream	-4161	ENSMUSG00000030125	Lrrc23
chr2	96362429	96362664	51,18	inside	204056	ENSMUSG00000050587	Lrrc4c
chr15	66296024	66296437	66,66	inside	36448	ENSMUSG00000022375	Lrrc6
chr2	30086765	30087227	58,35	upstream	-6470	ENSMUSG00000007476	Lrrc8a
chr9	111101919	111102179	54,67	inside	6374	ENSMUSG00000032497	Lrrfip2
chr3	155098461	155098776	55,49	downstream	342063	ENSMUSG00000028182	Lrriq3
chr1	127817304	127817696	55,23	upstream	-7626	ENSMUSG00000026344	Lypd1
chr2	49630853	49631200	54,76	upstream	-12355	ENSMUSG00000026765	Lypd6b
chr12	120792506	120792835	117,33	downstream	110623	ENSMUSG00000041886	Macc1
chr2	142068894	142069275	52,43	downstream	66551	ENSMUSG00000068205	Macrod2
chr2	160227668	160227890	54,86	upstream	-34867	ENSMUSG00000074622	Mafb
chr6	94157723	94157994	55,24	inside	75593	ENSMUSG00000045095	Magi1
chr5	19485387	19485674	52,70	inside	71708	ENSMUSG00000040003	Magi2
chr18	65615320	65615764	55,65	inside	24669	ENSMUSG00000032688	Malt1
chr9	13422445	13423331	1402,27	upstream	-1989	ENSMUSG00000031925	Maml2
chr10	19761865	19762391	67,81	upstream	-57466	ENSMUSG00000071369	Map3k5
chr18	4305370	4305660	51,97	downstream	30923	ENSMUSG00000024235	Map3k8
chr1	40077467	40077777	52,08	inside	58671	ENSMUSG00000026074	Map4k4
chr5	103503755	103504140	63,63	upstream	-16350	ENSMUSG00000046709	Mapk10
chr11	105317964	105318267	51,26	overlapStart	85	ENSMUSG00000078627	March10
chr10	36855860	36856156	50,09	inside	2872	ENSMUSG00000069662	Marcks
chr1	186771746	186772174	69,72	inside	51682	ENSMUSG00000026620	Mark1
chr14	120569007	120569364	50,78	upstream	-105884	ENSMUSG00000022139	Mbnl2
chr10	53342552	53342852	54,34	inside	7693	ENSMUSG00000058298	Mcm9
chr13	76980321	76980635	55,74	downstream	15577	ENSMUSG00000021596	Mctp1
chr6	16147204	16147494	51,47	downstream	469405	ENSMUSG00000041390	Mdfic
chr10	117518843	117519240	62,11	upstream	-60000	ENSMUSG00000020212	Mdm1
chr9	86582643	86582961	54,78	upstream	-1437	ENSMUSG00000032418	Me1
chr5	118986748	118987170	62,29	upstream	-23980	ENSMUSG00000018076	Med13l
chr13	83037222	83037587	57,85	upstream	-605811	ENSMUSG00000005583	Mef2c
chr5	104792173	104792554	109,68	downstream	37825	ENSMUSG00000053863	Mepe
chr8	63204108	63204413	53,13	downstream	69771	ENSMUSG00000031647	Mfap3l
chr7	143034968	143035345	57,54	upstream	-126906	ENSMUSG00000031004	Mki67
chr9	7608853	7609196	63,09	upstream	-19378	ENSMUSG00000018620	Mmp20
chr1	175799631	175799975	111,77	downstream	43422	ENSMUSG00000026536	Mnda
chr1	78576402	78576724	72,47	downstream	42199	ENSMUSG00000012187	Mogat1
chr1	72157343	72157636	51,25	downstream	101538	ENSMUSG00000039395	Mreg
chr7	55948818	55949105	62,72	upstream	-49647	ENSMUSG00000070546	Mrgprb3

chr11	5616372	5616848	50,48	upstream	-689	ENSMUSG00000020477	Mrps24
chr13	119105744	119106097	56,82	downstream	70315	ENSMUSG00000021731	Mrps30
chr19	11428233	11428564	51,77	upstream	-17597	ENSMUSG00000024672	Ms4a7
chr17	88084418	88084698	81,20	inside	12533	ENSMUSG00000024151	Msh2
chr13	93043411	93043799	70,79	upstream	-32354	ENSMUSG00000014850	Msh3
chr8	40999373	40999646	51,31	upstream	-271341	ENSMUSG00000025044	Msr1
chr8	40898297	40898626	87,94	upstream	-170265	ENSMUSG00000025044	Msr1
chr8	40780336	40780601	53,35	upstream	-52304	ENSMUSG00000025044	Msr1
chr14	65012416	65012741	62,73	inside	47406	ENSMUSG00000054733	Msra
chr3	82186278	82186624	57,59	inside	24284	ENSMUSG00000033900	Mtap9
chr2	90683938	90684293	55,02	upstream	-3374	ENSMUSG00000027282	Mtch2
chr9	89287415	89287740	53,14	downstream	174123	ENSMUSG00000066442	Mthfs
chr6	35435645	35435800	150,53	downstream	54243	ENSMUSG00000029840	Mtpn
chr2	110508920	110509239	64,29	upstream	-52577	ENSMUSG00000050808	Muc15
chr14	103641283	103641644	53,89	upstream	-50451	ENSMUSG00000033004	Mycbp2
chr14	103755340	103755690	86,94	upstream	-9309	ENSMUSG00000033004	Mycbp2
chrX	5060344	5060605	76,04	upstream	-13961	ENSMUSG00000044597	Mycs
chr11	68524415	68524984	64,08	inside	18597	ENSMUSG00000020900	Myh10
chr16	34724751	34725049	54,83	upstream	-20545	ENSMUSG00000022836	Mylk
chr13	32763423	32763776	55,98	downstream	110162	ENSMUSG00000044951	Mylk4
chr1	51838854	51839174	59,09	overlapStart	194	ENSMUSG00000018417	Myo1b
chr11	65057696	65057931	51,18	inside	25627	ENSMUSG00000020542	Myocd
chr8	24014786	24015110	53,37	upstream	-9472	ENSMUSG00000031540	Myst3
chr5	66226831	66227135	53,37	downstream	10350	ENSMUSG00000037795	N4bp2
chr5	97803761	97803995	51,44	downstream	17588	ENSMUSG00000046000	Naa11
chrX	100393033	100393232	87,04	upstream	-11054	ENSMUSG00000082229	Nap1l2
chr5	21211834	21212205	80,57	upstream	-4620	ENSMUSG00000044968	Napepld
chr7	104176713	104177009	50,53	inside	4534	ENSMUSG00000018995	Nars2
chr3	55987738	55988097	75,17	upstream	-115	ENSMUSG00000027799	Nbea
chr9	49366872	49367188	55,25	inside	11158	ENSMUSG00000039542	Ncam1
chr7	4303949	4304387	136,03	downstream	14578	ENSMUSG00000062524	Ncr1
chr3	123284108	123284526	83,37	upstream	-4675	ENSMUSG00000027977	Ndst3
chr3	125367027	125367394	52,89	downstream	259817	ENSMUSG00000027971	Ndst4
chr2	52100026	52100278	51,72	inside	11650	ENSMUSG00000026950	Neb
chr13	41609472	41609783	51,72	upstream	-26783	ENSMUSG00000021365	Nedd9
chr8	54772121	54772391	51,10	upstream	-47702	ENSMUSG00000039396	Neil3
chr14	16007041	16007363	58,59	downstream	243952	ENSMUSG00000042567	Nek10
chr7	57577696	57577984	52,46	downstream	224016	ENSMUSG00000055409	Nell1
chr18	81011123	81011642	69,53	upstream	-101313	ENSMUSG00000033016	Nfatc1
chr3	102303611	102303972	50,78	inside	29760	ENSMUSG00000027859	Ngf
chr15	34558233	34558488	56,02	inside	50228	ENSMUSG00000038879	Nipal2
chr15	8431880	8432180	54,34	upstream	-37417	ENSMUSG00000022141	Nipbl
chr3	25748443	25749008	114,71	inside	284213	ENSMUSG00000063887	Nlgn1
chr12	17445480	17445813	51,94	downstream	90195	ENSMUSG00000061458	Nol10
chr12	47873264	47873585	54,07	inside	46498	ENSMUSG00000021047	Nova1
chr17	4185427	4185767	54,33	upstream	-489166	ENSMUSG00000023802	Nox3
chr9	23803959	23804176	50,97	upstream	-98481	ENSMUSG00000043659	Npsr1
chr9	23874264	23874580	55,25	upstream	-28176	ENSMUSG00000043659	Npsr1
chr6	50705791	50706134	73,09	upstream	-101353	ENSMUSG00000029831	Npvf
chr14	40176958	40177272	55,74	inside	109130	ENSMUSG00000041014	Nrg3
chr15	59225330	59225705	66,11	inside	19577	ENSMUSG00000059586	Nsmce2
chr12	10127426	10127778	56,20	upstream	-249353	ENSMUSG00000020622	Nt5c1b
chr10	117223569	117223981	51,81	inside	6185	ENSMUSG00000052798	Nup107
chr6	9446696	9446968	51,56	downstream	547020	ENSMUSG00000046178	Nxph1
chr2	23148656	23148997	63,59	upstream	-28110	ENSMUSG00000069132	Nxph2
chr1	51583278	51583697	109,71	upstream	-48035	ENSMUSG00000026107	Obfc2a
chr7	63584032	63584336	63,22	downstream	22424	ENSMUSG00000030450	Oca2
chr12	17486580	17486893	53,19	upstream	-65099	ENSMUSG00000011179	Odc1
chr11	37055482	37055862	50,19	upstream	-6016	ENSMUSG00000049336	Odz2
chr8	49816232	49816550	59,59	upstream	-56188	ENSMUSG00000031561	Odz3
chr13	40397196	40397589	51,83	upstream	-13816	ENSMUSG00000047094	Ofcc1
chr11	6215249	6215517	52,57	inside	22641	ENSMUSG00000020456	Ogdh
chr17	37874825	37875242	72,08	upstream	-10986	ENSMUSG00000060017	Olfr121
chr2	88979449	88979748	64,58	downstream	5729	ENSMUSG00000075100	Olfr1223
chr13	21703297	21703738	77,24	inside	576	ENSMUSG00000051258	Olfr1362
chr9	38222757	38223119	143,52	downstream	13452	ENSMUSG00000049098	Olfr147
chr19	13636247	13636574	52,68	upstream	-23549	ENSMUSG00000057213	Olfr1484

chr9	37514451	37514775	53,37	downstream	5411	ENSMUSG00000061165	Olfr151
chr16	59231985	59232242	50,43	upstream	-15544	ENSMUSG00000074996	Olfr199
chr16	59244393	59244716	125,95	downstream	25098	ENSMUSG00000074995	Olfr201
chr16	59243538	59243827	96,54	downstream	25953	ENSMUSG00000074995	Olfr201
chr16	59370212	59370486	56,01	upstream	-8170	ENSMUSG00000045504	Olfr209
chr1	176056781	176057178	64,37	upstream	-8636	ENSMUSG00000046486	Olfr231
chr7	93759795	93760100	53,13	downstream	5358	ENSMUSG00000062042	Olfr294
chr2	37168703	37168864	105,88	upstream	-18566	ENSMUSG00000049018	Olfr368
chr1	176072684	176072956	51,56	downstream	5807	ENSMUSG00000051528	Olfr424
chr6	43070810	43071128	54,78	downstream	5112	ENSMUSG00000073109	Olfr441
chr14	54935539	54935719	60,06	upstream	-32939	ENSMUSG00000048153	Olfr49
chr4	43723534	43723875	51,42	upstream	-431	ENSMUSG00000046450	Olfr71
chr14	50649062	50649400	59,49	downstream	6113	ENSMUSG00000068437	Olfr725
chr10	129101745	129102072	52,68	downstream	4885	ENSMUSG00000063452	Olfr800
chr10	129575698	129576006	52,42	upstream	-11587	ENSMUSG00000057264	Olfr824
chr9	19279984	19280299	50,80	downstream	2708	ENSMUSG00000057985	Olfr850
chr9	19937460	19937755	50,77	inside	3896	ENSMUSG00000058491	Olfr869
chr10	19147234	19147534	54,34	downstream	70868	ENSMUSG00000045591	Olig3
chr10	19178877	19179278	58,91	downstream	102511	ENSMUSG00000045591	Olig3
chr11	79318618	79318893	50,81	upstream	-1034	ENSMUSG00000049612	Omg
chr9	28376453	28376893	59,44	upstream	-92761	ENSMUSG00000062257	Opcml
chr16	87571618	87571982	52,43	inside	18043	ENSMUSG00000025610	ORF63
chr9	115058361	115059024	166,67	inside	81964	ENSMUSG00000040875	Osbpl10
chr9	115059248	115060239	543,76	inside	82851	ENSMUSG00000040875	Osbpl10
chr9	115060529	115061663	407,53	inside	84132	ENSMUSG00000040875	Osbpl10
chr6	50376207	50376495	62,44	upstream	-29056	ENSMUSG00000029822	Osbpl3
chr3	69915789	69916047	137,15	downstream	104254	ENSMUSG00000027788	Otol1
chr4	133967492	133967898	55,33	inside	8607	ENSMUSG00000037366	Pafah2
chr4	64861008	64861286	50,08	inside	75800	ENSMUSG00000028370	Pappa
chr1	160725280	160725562	51,47	inside	19398	ENSMUSG00000073530	Pappa2
chr17	11577119	11577419	59,24	downstream	146794	ENSMUSG00000023826	Park2
chr6	39033686	39034004	54,78	downstream	7406	ENSMUSG00000038507	Parp12
chr2	122002230	122002520	51,97	inside	9695	ENSMUSG00000027233	Patl2
chr10	107741140	107741467	57,37	upstream	-28105	ENSMUSG00000035873	Pawr
chr10	107742115	107742363	63,36	upstream	-27130	ENSMUSG00000035873	Pawr
chr1	78099888	78100256	61,35	inside	93823	ENSMUSG00000004872	Pax3
chr4	139352085	139352562	60,70	inside	36798	ENSMUSG00000028736	Pax7
chr14	94404014	94404417	72,42	upstream	-116063	ENSMUSG00000055421	Pcdh9
chr5	14673547	14673833	52,95	inside	158629	ENSMUSG00000061601	Pclo
chr1	7133734	7134065	56,42	upstream	-22808	ENSMUSG00000051285	Pcmtd1
chr6	141333738	141334125	70,79	inside	135225	ENSMUSG00000041741	Pde3a
chr4	101797579	101797931	56,20	inside	37431	ENSMUSG00000028525	Pde4b
chr13	109288581	109288904	53,60	inside	48378	ENSMUSG00000021699	Pde4d
chr6	136907765	136908081	65,09	overlapStart	-17	ENSMUSG00000064330	Pde6h
chr10	20508522	20509001	52,28	upstream	-63638	ENSMUSG00000019990	Pde7b
chr15	92571918	92572171	51,46	inside	144378	ENSMUSG00000036218	Pdzrn4
chr3	57629503	57629719	56,66	downstream	20297	ENSMUSG00000027805	Pfn2
chr2	156106975	156107253	50,08	upstream	-1392	ENSMUSG00000038116	Phf20
chr16	45768063	45768304	53,76	inside	4333	ENSMUSG00000033149	Phldb2
chr16	45880531	45880808	55,23	downstream	21549	ENSMUSG00000033149	Phldb2
chr9	62770679	62770976	55,08	inside	30167	ENSMUSG00000032405	Pias1
chr1	84441915	84442240	62,73	upstream	-160695	ENSMUSG00000045658	Pid1
chr13	102656043	102656428	77,03	upstream	-117871	ENSMUSG00000041417	Pik3r1
chr1	55643880	55644206	57,62	upstream	-108707	ENSMUSG00000038349	Plcl1
chr1	55999174	55999507	51,33	downstream	246587	ENSMUSG00000038349	Plcl1
chr17	50545933	50546203	52,06	upstream	-102939	ENSMUSG00000038910	Plcl2
chr2	146799259	146799533	51,06	downstream	20640	ENSMUSG00000074749	Plk1s1
chr1	196529316	196529638	58,02	downstream	58266	ENSMUSG00000026640	Plxna2
chr3	10180201	10180510	61,10	inside	3684	ENSMUSG00000052468	Pmp2
chr12	45385357	45385634	50,32	inside	1797	ENSMUSG00000036257	Pnpla8
chr10	105775311	105775767	258,88	upstream	-127010	ENSMUSG00000053825	Ppfia2
chr2	69587719	69588117	99,56	downstream	7993	ENSMUSG00000042133	Ppig
chr12	70277019	70277251	52,00	inside	7197	ENSMUSG00000034883	Ppil5
chr12	73815902	73816170	52,57	upstream	-46296	ENSMUSG00000021096	Ppm1a
chr6	57456712	57456989	50,32	inside	28708	ENSMUSG00000037826	Ppm1k
chr7	107501313	107501588	66,13	inside	19093	ENSMUSG00000030718	Ppme1
chr9	101095292	101095645	51,45	inside	6111	ENSMUSG00000043154	Ppp2r3a

chr3	136164488	136164889	77,51	upstream	-168600	ENSMUSG00000028161	Ppp3ca
chr18	53664958	53665215	55,48	inside	32323	ENSMUSG00000069378	Prdm6
chr18	41887966	41888287	58,84	downstream	222882	ENSMUSG00000056671	Prelid2
chr1	152339154	152339473	59,34	upstream	-25859	ENSMUSG00000006014	Prg4
chr15	93493110	93493432	55,14	upstream	-66788	ENSMUSG00000036158	Prickle1
chr1	33724660	33725054	65,07	inside	1971	ENSMUSG00000026134	Prim2
chr12	74789516	74789882	54,77	inside	103686	ENSMUSG00000021108	Prkch
chr12	51530198	51530580	58,59	inside	220012	ENSMUSG00000002688	Prkd1
chr13	13000727	13001620	51,81	downstream	96874	ENSMUSG00000079092	Prl2c2
chr13	12937237	12937684	65,29	upstream	-44892	ENSMUSG00000056457	Prl2c3
chr16	63040836	63041197	73,32	downstream	119543	ENSMUSG00000022912	Pros1
chr7	18969224	18969716	76,88	upstream	-28868	ENSMUSG00000003505	Psg18
chr2	61567352	61567642	51,97	inside	6581	ENSMUSG00000026914	Psmd14
chr4	75989965	75990356	69,20	overlapStart	160	ENSMUSG00000028399	Ptprd
chr4	77727403	77727629	64,69	inside	130240	ENSMUSG00000028399	Ptprd
chr14	12368728	12369009	53,20	upstream	-17318	ENSMUSG00000021745	Ptprg
chr17	67569072	67569365	55,61	inside	134341	ENSMUSG00000033278	Ptprm
chr12	30707271	30707542	50,54	downstream	22591	ENSMUSG00000020674	Pxdn
chr3	5630437	5630804	66,82	upstream	-54198	ENSMUSG00000040374	Pxmp3
chr1	175508187	175508872	67,65	inside	21018	ENSMUSG00000073491	Pydc4
chr4	11521697	11522055	59,46	upstream	-14825	ENSMUSG00000078773	Rad54b
chr2	146261230	146261590	63,72	inside	11798	ENSMUSG00000037110	Ralgapa2
chr14	120862600	120862959	52,43	upstream	-15066	ENSMUSG00000051615	Rap2a
chr12	118777803	118778165	53,97	inside	22851	ENSMUSG00000041992	Rapgef5
chr11	54507271	54507564	51,25	inside	2337	ENSMUSG00000037533	Rapgef6
chr13	85536457	85536785	57,13	upstream	-107366	ENSMUSG00000021549	Rasa1
chr17	75861236	75861547	51,72	upstream	-3642	ENSMUSG00000071042	Rasgrp3
chr5	91126832	91127211	51,89	upstream	-57319	ENSMUSG00000029370	Rassf6
chr14	73700429	73700688	60,13	upstream	-12915	ENSMUSG00000022105	Rb1
chr18	11471013	11471287	51,06	upstream	-320772	ENSMUSG00000041238	Rbbp8
chr18	11781340	11781768	75,89	upstream	-10445	ENSMUSG00000041238	Rbbp8
chr16	7211958	7212280	63,51	upstream	-6388	ENSMUSG00000008658	Rbfox1
chr8	93601057	93601357	54,34	inside	7064	ENSMUSG00000031666	Rbl2
chr9	117213248	117213590	52,79	inside	48435	ENSMUSG00000039607	Rbms3
chr9	116680863	116681195	60,96	downstream	479830	ENSMUSG00000039607	Rbms3
chr5	92280235	92280642	62,10	downstream	102545	ENSMUSG00000029397	Rchy1
chr1	16106114	16106426	55,84	inside	10259	ENSMUSG00000025921	Rdh10
chr6	71672919	71673291	51,91	inside	15364	ENSMUSG00000052852	Reep1
chr6	78420218	78420499	59,22	upstream	-1350	ENSMUSG00000030017	Reg3g
chr6	137069494	137069842	61,35	inside	48712	ENSMUSG00000030222	Rerg
chr10	39596632	39597011	52,39	downstream	9717	ENSMUSG00000019841	Rev3l
chr10	84215313	84215812	62,58	upstream	-3480	ENSMUSG00000020037	Rfx4
chr10	4086659	4086962	56,21	upstream	-337482	ENSMUSG00000019775	Rgs17
chr12	83789205	83789498	51,25	inside	36389	ENSMUSG00000021219	Rgs6
chr10	68629734	68629999	52,38	inside	15552	ENSMUSG00000019944	Rhobtb1
chr11	80017584	80017895	51,47	upstream	-4937	ENSMUSG00000017686	Rhot1
chr15	6718076	6718396	54,30	inside	59693	ENSMUSG00000050310	Rictor
chr14	62986991	62987433	52,93	downstream	20855	ENSMUSG00000021932	Rnaseh2b
chr11	79998981	79999359	115,17	inside	1256	ENSMUSG00000020707	Rnf135
chr13	47307019	47307404	62,51	inside	17647	ENSMUSG00000038068	Rnf144b
chr11	44325617	44325869	51,72	upstream	-6849	ENSMUSG00000019189	Rnf145
chr1	39617737	39618028	51,73	upstream	-2135	ENSMUSG00000048234	Rnf149
chr13	106133888	106134284	51,23	upstream	-72769	ENSMUSG00000021720	Rnf180
chr13	106057550	106057914	67,57	inside	3569	ENSMUSG00000021720	Rnf180
chr18	68483540	68483836	50,53	inside	4889	ENSMUSG00000009535	Rnmt
chr16	72265179	72265557	56,82	upstream	-398215	ENSMUSG00000022883	Robo1
chr4	59503931	59504231	54,34	downstream	23289	ENSMUSG00000028382	Rod1
chr16	5021308	5021693	57,93	upstream	-7688	ENSMUSG00000022540	Rogdi
chr9	68964357	68964635	54,97	upstream	-78991	ENSMUSG00000032238	Rora
chr19	18995855	18996033	378,46	downstream	79735	ENSMUSG00000036192	Rorb
chr14	64623078	64623407	57,39	inside	11810	ENSMUSG00000046049	Rp1l1
chr14	3009789	3009961	113,10	upstream	-20213	ENSMUSG00000090487	RP24-211P18.1
chr14	25370100	25370440	54,33	downstream	60160	ENSMUSG00000025290	Rps24
chr11	86333908	86334266	59,46	upstream	-367	ENSMUSG00000020516	Rps6kb1
chr3	149497118	149497360	54,43	downstream	239466	ENSMUSG00000047676	Rpsa-ps10
chr12	27146280	27146625	66,24	upstream	-4963	ENSMUSG00000020641	Rsad2
chr17	44886850	44887123	51,31	upstream	-13253	ENSMUSG00000039153	Runx2

chr2	112857270	112857643	78,83	inside	13218	ENSMUSG00000057378	Ryr3
chr14	61747799	61748107	52,42	upstream	-9495	ENSMUSG00000048279	Sacs
chr16	76030180	76030566	74,93	upstream	-7665	ENSMUSG00000022876	Samsn1
chr16	90310628	90310969	54,10	upstream	-25958	ENSMUSG00000022983	Scaf4
chr16	90274310	90274629	59,34	upstream	-9487	ENSMUSG00000022983	Scaf4
chr1	181607221	181607646	62,29	upstream	-3428	ENSMUSG00000038936	Sccpdh
chr2	65564696	65565057	54,19	downstream	9086	ENSMUSG00000075318	Scn2a1
chr8	59906635	59906975	59,01	upstream	-28085	ENSMUSG00000031610	Scrg1
chr1	165832220	165832495	50,81	upstream	-27011	ENSMUSG00000026584	Scyl3
chr1	51427487	51427741	51,20	downstream	81517	ENSMUSG00000045954	Sdpr
chr1	159851476	159851873	69,08	downstream	383294	ENSMUSG00000026589	Sec16b
chr3	30715921	30716258	85,44	inside	24123	ENSMUSG00000027706	Sec62
chr5	17070512	17070818	52,89	upstream	-9587	ENSMUSG00000028780	Sema3c
chr18	47704528	47704862	65,37	upstream	-176004	ENSMUSG00000019647	Sema6a
chr12	105206758	105207083	50,50	upstream	-9573	ENSMUSG00000072849	Serpina1e
chr12	105242853	105243200	81,54	inside	9009	ENSMUSG00000058260	Serpina9
chr13	33988343	33988681	50,23	inside	8696	ENSMUSG00000052180	Serpinb6c
chr3	66759083	66759484	98,65	downstream	23271	ENSMUSG00000027833	Shox2
chr3	66680824	66681109	68,55	downstream	101530	ENSMUSG00000027833	Shox2
chr8	5148011	5148340	61,71	upstream	-42779	ENSMUSG00000023073	Slc10a2
chr16	33603385	33603667	69,44	inside	6672	ENSMUSG00000035506	Slc12a8
chr10	124594891	124595247	59,93	downstream	150981	ENSMUSG00000020102	Slc16a7
chr7	58927006	58927306	64,30	downstream	49806	ENSMUSG00000030500	Slc17a6
chr15	8631060	8631400	68,81	downstream	28528	ENSMUSG00000005360	Slc1a3
chr6	6089916	6090238	107,75	inside	52689	ENSMUSG00000015112	Slc25a13
chr15	81149899	81150279	66,22	inside	41296	ENSMUSG00000022404	Slc25a17
chr12	57938008	57938418	75,53	upstream	-61661	ENSMUSG00000035472	Slc25a21
chr5	8451300	8451612	51,47	inside	28312	ENSMUSG00000054099	Slc25a40
chr13	58727305	58727601	51,95	upstream	-15067	ENSMUSG00000021553	Slc28a3
chr13	58613241	58613558	52,05	downstream	46387	ENSMUSG00000021553	Slc28a3
chr2	165368136	165368609	52,74	downstream	33855	ENSMUSG00000027661	Slc2a10
chr15	91291395	91291688	51,47	upstream	-48747	ENSMUSG00000036298	Slc2a13
chr5	67740083	67740476	60,68	inside	16726	ENSMUSG00000029221	Slc30a9
chr1	127469504	127469794	55,80	inside	11911	ENSMUSG00000026342	Slc35f5
chr13	113515625	113515940	50,80	upstream	-8236	ENSMUSG00000069056	Slc38a9
chr1	46914841	46915098	50,43	upstream	-3950	ENSMUSG00000025986	Slc39a10
chr6	141419259	141419655	53,89	upstream	-53648	ENSMUSG00000030235	Slco1c1
chr7	81772986	81773333	56,22	upstream	-73320	ENSMUSG00000025790	Slco3a1
chr7	81691877	81692112	57,61	inside	5603	ENSMUSG00000025790	Slco3a1
chr7	81686734	81687161	62,20	inside	10746	ENSMUSG00000025790	Slco3a1
chr5	48018292	48018610	50,12	upstream	-356102	ENSMUSG00000031558	Slit2
chr11	35332537	35332858	54,94	upstream	-25650	ENSMUSG00000056427	Slit3
chr19	26694137	26694498	61,59	inside	12377	ENSMUSG00000024921	Smarca2
chr19	26728656	26729011	55,52	inside	46896	ENSMUSG00000024921	Smarca2
chr9	110055435	110055793	59,46	inside	20894	ENSMUSG00000032481	Smarcc1
chr17	13384021	13384383	52,45	upstream	-30033	ENSMUSG00000073458	Smok2a
chr4	40693454	40693778	58,10	downstream	10977	ENSMUSG00000028409	Smu1
chr1	181423531	181423809	50,08	inside	24551	ENSMUSG00000055067	Smyd3
chr14	14483589	14483906	55,01	upstream	-19801	ENSMUSG00000044772	Sntn
chr3	117442709	117443088	59,27	downstream	129030	ENSMUSG00000028007	Snx7
chr9	41979817	41980195	50,59	upstream	-47437	ENSMUSG00000049313	Sorl1
chr7	123192592	123192879	52,70	upstream	-10334	ENSMUSG00000051910	Sox6
chr7	123146201	123146578	50,80	upstream	-7601	ENSMUSG00000051910	Sox6
chr7	122987954	122988365	65,78	inside	1665	ENSMUSG00000051910	Sox6
chr1	70072815	70073113	51,47	inside	29439	ENSMUSG00000053153	Spag16
chr1	70224074	70224401	57,37	inside	180698	ENSMUSG00000053153	Spag16
chr1	70335915	70336171	50,68	downstream	292539	ENSMUSG00000053153	Spag16
chr16	69576233	69576556	53,60	downstream	284498	ENSMUSG00000063163	Speer2
chr5	17013876	17014242	82,24	downstream	31960	ENSMUSG00000058643	Speer4f
chr14	75950364	75950732	57,17	downstream	41428	ENSMUSG00000034913	Spert
chr11	30253570	30253874	58,22	upstream	-85395	ENSMUSG00000020315	Spnb2
chr7	121023145	121023555	57,84	inside	113457	ENSMUSG00000038156	Spon1
chr5	77424329	77424617	52,46	inside	47	ENSMUSG00000036323	Srp72
chr13	91767032	91767445	79,68	upstream	-43010	ENSMUSG00000003992	Ssbp2
chr4	106658740	106659253	64,98	inside	19709	ENSMUSG00000061887	Ssbp3
chr13	38083210	38083563	51,88	inside	2784	ENSMUSG00000021427	Ssr1
chrX	8306121	8306372	90,35	upstream	-19437	ENSMUSG00000035371	Ssx9

chr1	97568511	97568824	60,86	upstream	-4340	ENSMUSG00000040710	St8sia4
chr10	86399855	86400222	56,87	inside	3530	ENSMUSG00000035459	Stab2
chr9	100563414	100563692	50,08	inside	19225	ENSMUSG00000037286	Stag1
chr1	16543315	16543467	125,16	upstream	-33122	ENSMUSG00000025920	Stau2
chr1	16486448	16486923	65,90	inside	22844	ENSMUSG00000025920	Stau2
chr1	16464987	16465281	51,01	inside	44305	ENSMUSG00000025920	Stau2
chr15	35093827	35094078	51,99	upstream	-8266	ENSMUSG00000022329	Stk3
chr6	137677054	137677353	54,59	upstream	-6545	ENSMUSG00000030224	Strap
chr3	145657972	145658273	64,03	inside	7138	ENSMUSG00000036863	Syde2
chr10	7719719	7720177	73,45	upstream	-43691	ENSMUSG00000015755	Tab2
chr14	101875753	101876073	54,30	upstream	-13313	ENSMUSG00000033083	Tbc1d4
chr17	51464139	51464414	50,81	upstream	-145465	ENSMUSG00000023923	Tbc1d5
chr17	51310057	51310444	80,73	inside	8452	ENSMUSG00000023923	Tbc1d5
chr18	42673768	42674025	50,43	inside	2627	ENSMUSG00000024498	Tcerg1
chr18	42683634	42683984	69,89	inside	12493	ENSMUSG00000024498	Tcerg1
chr10	22663988	22664410	77,45	upstream	-124054	ENSMUSG00000045680	Tcf21
chr19	56913815	56914167	56,82	inside	8613	ENSMUSG00000025081	Tdrd1
chr14	87793677	87794121	97,69	upstream	-22713	ENSMUSG00000022019	Tdrd3
chr7	25458945	25459168	54,56	upstream	-1876	ENSMUSG00000062773	Tex101
chr7	25440647	25441023	59,37	downstream	16422	ENSMUSG00000062773	Tex101
chr17	3549457	3549729	56,54	inside	8256	ENSMUSG00000036983	Tfb1m
chr17	81451291	81451618	52,68	inside	13134	ENSMUSG00000024246	Thumpd2
chr9	76775774	76776037	53,87	downstream	117814	ENSMUSG00000032357	Tinag
chr19	41294577	41295029	80,47	upstream	-13313	ENSMUSG00000025013	Tll2
chr9	67470620	67470989	54,69	upstream	-63110	ENSMUSG00000052698	Tln2
chr9	67198543	67198788	51,84	upstream	-23072	ENSMUSG00000052698	Tln2
chr3	57248136	57248512	64,63	downstream	18900	ENSMUSG00000027801	Tm4sf4
chr15	39604550	39604831	59,22	downstream	18807	ENSMUSG00000022303	Tm7sf4
chr13	55686117	55686428	51,47	upstream	-8196	ENSMUSG00000058569	Tmed9
chr1	50985760	50986071	51,72	inside	968	ENSMUSG00000026109	Tmeff2
chr5	127564825	127565142	50,34	upstream	-157353	ENSMUSG00000034324	Tmem132c
chr7	96023815	96024102	52,70	downstream	278995	ENSMUSG00000039428	Tmem135
chr1	41014699	41015073	65,11	downstream	152253	ENSMUSG00000079588	Tmem182
chr1	52705559	52705825	63,42	inside	6731	ENSMUSG00000043015	Tmem194b
chr12	38244727	38245067	54,33	inside	276098	ENSMUSG00000050103	Tmem195
chr6	30436504	30436901	64,37	inside	22010	ENSMUSG00000029782	Tmem209
chr9	31306778	31307074	50,53	upstream	-34955	ENSMUSG00000041737	Tmem45b
chr5	20395348	20395687	54,56	downstream	7339	ENSMUSG00000045435	Tmem60
chr10	99946108	99946385	56,21	inside	3793	ENSMUSG00000036676	Tmtc3
chr1	73387614	73387939	53,14	upstream	-325141	ENSMUSG00000026182	Tnp1
chr1	73325687	73325982	50,77	upstream	-263214	ENSMUSG00000026182	Tnp1
chr13	99722030	99722332	74,28	upstream	-25691	ENSMUSG00000009470	Tnpo1
chr10	39355580	39356009	54,44	upstream	-5360	ENSMUSG00000019842	Traf3ip2
chr12	15684881	15685178	55,08	downstream	138710	ENSMUSG00000020601	Trib2
chr4	65034861	65035226	52,43	upstream	-231159	ENSMUSG00000051675	Trim32
chr11	87034354	87034776	77,38	downstream	30778	ENSMUSG00000018548	Trim37
chr8	127305618	127306088	50,21	upstream	-11301	ENSMUSG00000036913	Trim67
chr16	25659362	25659694	60,96	upstream	-24487	ENSMUSG00000022510	Trp63
chr19	22237714	22237970	50,68	inside	24105	ENSMUSG00000052387	Trpm3
chr15	51077458	51077827	52,43	upstream	-355871	ENSMUSG00000038679	Trps1
chr18	84052267	84052586	64,29	downstream	203687	ENSMUSG00000046982	Tshz1
chr3	33631702	33632022	59,09	upstream	-67175	ENSMUSG00000027677	Ttc14
chr15	10472384	10472672	51,47	upstream	-11927	ENSMUSG00000022249	Ttc23l
chr8	80794780	80795084	53,37	inside	19390	ENSMUSG00000037101	Ttc29
chr12	87227427	87227742	50,80	inside	60028	ENSMUSG00000012609	Ttll5
chr11	96014386	96014734	51,47	upstream	-3404	ENSMUSG00000038756	Ttll6
chr3	146941310	146941669	59,23	downstream	334299	ENSMUSG00000036745	Ttll7
chr2	76807552	76807973	63,49	inside	10687	ENSMUSG00000051747	Ttn
chr17	65984187	65984502	53,42	downstream	7357	ENSMUSG00000050612	Txndc2
chr14	19733551	19733941	56,83	upstream	-6770	ENSMUSG00000058317	Ube2e2
chr7	68781220	68781585	56,82	upstream	-86633	ENSMUSG00000059585	Ube2nl
chr7	66325509	66325843	55,71	upstream	-158613	ENSMUSG00000025326	Ube3a
chr1	36285206	36285681	53,90	inside	15941	ENSMUSG00000037470	Uggt1
chr17	31108773	31109120	55,45	inside	17145	ENSMUSG00000054134	Umodl1
chr16	33968266	33968640	51,42	upstream	-1142	ENSMUSG00000022814	Umps
chr8	31035633	31035894	54,40	upstream	-705823	ENSMUSG00000063626	Unc5d
chr8	30401171	30401503	51,55	upstream	-71361	ENSMUSG00000063626	Unc5d

chr8	29491523	29491871	52,54	downstream	510463	ENSMUSG00000063626	Unc5d
chr2	155742871	155743216	53,20	upstream	-6531	ENSMUSG00000005882	Uqcc
chr11	79754091	79754402	61,38	inside	1675	ENSMUSG00000035575	Utp6
chr10	12231684	12231985	58,99	inside	102748	ENSMUSG00000019820	Utrn
chr3	102049812	102050360	59,93	upstream	-41196	ENSMUSG00000027860	Vangl1
chr10	4692993	4693326	109,75	downstream	14330	ENSMUSG00000019772	Vip
chr13	23188774	23189145	56,50	upstream	-2166	ENSMUSG00000057799	Vmn1r216
chr7	12791520	12791822	51,47	downstream	13206	ENSMUSG00000057161	Vmn1r80
chr5	109442491	109442911	98,36	upstream	-7036	ENSMUSG00000067010	Vmn2r10
chrX	121324851	121325168	190,44	upstream	-75332	ENSMUSG00000072049	Vmn2r121
chrX	121321752	121321974	117,29	upstream	-72233	ENSMUSG00000072049	Vmn2r121
chrX	121320712	121321068	206,11	upstream	-71193	ENSMUSG00000072049	Vmn2r121
chrX	121315380	121315736	287,31	upstream	-65861	ENSMUSG00000072049	Vmn2r121
chrX	121314335	121315123	355,44	upstream	-64816	ENSMUSG00000072049	Vmn2r121
chrX	121313932	121314254	392,76	upstream	-64413	ENSMUSG00000072049	Vmn2r121
chrX	121312707	121313253	206,94	upstream	-63188	ENSMUSG00000072049	Vmn2r121
chrX	121311525	121311911	200,12	upstream	-62006	ENSMUSG00000072049	Vmn2r121
chrX	121311144	121311513	306,84	upstream	-61625	ENSMUSG00000072049	Vmn2r121
chrX	121310499	121310653	93,38	upstream	-60980	ENSMUSG00000072049	Vmn2r121
chrX	121306452	121309177	3100,00	upstream	-56933	ENSMUSG00000072049	Vmn2r121
chrX	121304210	121305045	856,36	upstream	-54691	ENSMUSG00000072049	Vmn2r121
chrX	121303111	121304072	1263,24	upstream	-53592	ENSMUSG00000072049	Vmn2r121
chrX	121302656	121302978	188,85	upstream	-53137	ENSMUSG00000072049	Vmn2r121
chrX	121302025	121302354	337,89	upstream	-52506	ENSMUSG00000072049	Vmn2r121
chrX	121300760	121301935	988,79	upstream	-51241	ENSMUSG00000072049	Vmn2r121
chrX	121299312	121300160	791,02	upstream	-49793	ENSMUSG00000072049	Vmn2r121
chrX	121297352	121297636	53,45	upstream	-47833	ENSMUSG00000072049	Vmn2r121
chrX	121291736	121292393	462,82	upstream	-42217	ENSMUSG00000072049	Vmn2r121
chrX	121290779	121291073	166,30	upstream	-41260	ENSMUSG00000072049	Vmn2r121
chrX	121288602	121288916	80,91	upstream	-39083	ENSMUSG00000072049	Vmn2r121
chrX	121287485	121287798	66,66	upstream	-37966	ENSMUSG00000072049	Vmn2r121
chrX	121285796	121286089	82,63	upstream	-36277	ENSMUSG00000072049	Vmn2r121
chrX	121284749	121285030	137,61	upstream	-35230	ENSMUSG00000072049	Vmn2r121
chrX	121283920	121284125	125,24	upstream	-34401	ENSMUSG00000072049	Vmn2r121
chrX	121283658	121283881	131,04	upstream	-34139	ENSMUSG00000072049	Vmn2r121
chrX	121283083	121283375	80,73	upstream	-33564	ENSMUSG00000072049	Vmn2r121
chrX	121282339	121282840	171,46	upstream	-32820	ENSMUSG00000072049	Vmn2r121
chrX	121281352	121281606	61,57	upstream	-31833	ENSMUSG00000072049	Vmn2r121
chrX	121280493	121280906	69,99	upstream	-30974	ENSMUSG00000072049	Vmn2r121
chrX	121278107	121278658	602,96	upstream	-28588	ENSMUSG00000072049	Vmn2r121
chrX	121274709	121275198	86,22	upstream	-25190	ENSMUSG00000072049	Vmn2r121
chrX	121271807	121272136	107,28	upstream	-22288	ENSMUSG00000072049	Vmn2r121
chrX	121269476	121270408	380,60	upstream	-19957	ENSMUSG00000072049	Vmn2r121
chrX	121269088	121269394	312,82	upstream	-19569	ENSMUSG00000072049	Vmn2r121
chrX	121267872	121268649	528,88	upstream	-18353	ENSMUSG00000072049	Vmn2r121
chrX	121267148	121267578	221,56	upstream	-17629	ENSMUSG00000072049	Vmn2r121
chrX	121263788	121264676	433,41	upstream	-14269	ENSMUSG00000072049	Vmn2r121
chrX	121216944	121217185	469,94	downstream	32575	ENSMUSG00000072049	Vmn2r121
chrX	121216690	121216913	71,03	downstream	32829	ENSMUSG00000072049	Vmn2r121
chrX	121215653	121216212	455,37	downstream	33866	ENSMUSG00000072049	Vmn2r121
chrX	121214908	121215426	422,56	downstream	34611	ENSMUSG00000072049	Vmn2r121
chrX	121214679	121214842	112,53	downstream	34840	ENSMUSG00000072049	Vmn2r121
chrX	121214271	121214574	96,64	downstream	35248	ENSMUSG00000072049	Vmn2r121
chrX	121210504	121210802	264,40	downstream	39015	ENSMUSG00000072049	Vmn2r121
chrX	121210201	121210355	163,39	downstream	39318	ENSMUSG00000072049	Vmn2r121
chrX	121185504	121185745	60,03	downstream	64015	ENSMUSG00000072049	Vmn2r121
chr14	52082456	52082896	127,41	downstream	10778	ENSMUSG00000070448	Vmn2r89
chr10	23569884	23570240	75,70	upstream	-1384	ENSMUSG00000020010	Vnn3
chr19	16717871	16718269	58,03	inside	23047	ENSMUSG00000046230	Vps13a
chr13	8842423	8842759	50,67	downstream	27558	ENSMUSG00000021147	Wdr37
chr1	177646313	177646596	51,47	inside	17589	ENSMUSG00000026523	Wdr64
chr1	45861393	45861739	62,34	inside	862	ENSMUSG00000025995	Wdr75
chr11	17076532	17076845	51,26	upstream	-5578	ENSMUSG00000078970	Wdr92
chr6	22202120	22202416	58,28	upstream	-36107	ENSMUSG00000029671	Wnt16
chr6	17986054	17986383	52,43	upstream	-5469	ENSMUSG00000010797	Wnt2
chr2	67387847	67388125	50,08	downstream	32827	ENSMUSG00000027022	Xirp2
chrX	51097800	51098265	55,65	inside	3073	ENSMUSG00000054626	Xlr

chr19	52959852	52960109	50,43	downstream	153191	ENSMUSG00000025027	Xpnpep1
chr1	129716248	129716577	51,75	inside	20619	ENSMUSG00000051590	Ysk4
chr16	43619433	43619777	51,62	upstream	-1557	ENSMUSG00000036279	Zbtb20
chrX	93016149	93016317	135,62	upstream	-78286	ENSMUSG00000035045	Zc3h12b
chr9	52019632	52019922	51,97	upstream	-43416	ENSMUSG00000035164	Zc3h12c
chr14	58558822	58559148	57,62	upstream	-49723	ENSMUSG00000021969	Zdhhc20
chr15	67647117	67647427	61,64	downstream	443232	ENSMUSG00000022335	Zfat
chrX	51086123	51086492	162,76	upstream	-6552	ENSMUSG00000059334	Zfp36l3
chrX	51082807	51083191	121,27	upstream	-3236	ENSMUSG00000059334	Zfp36l3
chrX	51082032	51082200	55,69	upstream	-2461	ENSMUSG00000059334	Zfp36l3
chr6	47795799	47796125	56,51	inside	10105	ENSMUSG00000062519	Zfp398
chr18	84718006	84718288	53,95	inside	9761	ENSMUSG00000048410	Zfp407
chr9	20289121	20289498	61,35	inside	6128	ENSMUSG00000059475	Zfp426
chr9	65651438	65651717	64,97	upstream	-5719	ENSMUSG00000040524	Zfp609
chr9	65620098	65620376	53,97	downstream	25621	ENSMUSG00000040524	Zfp609
chr8	27829495	27829948	64,02	upstream	-258313	ENSMUSG00000085795	Zfp703
chr8	81699436	81699826	61,01	overlapStart	-273	ENSMUSG00000071064	Zfp827
chr15	40779551	40779847	50,53	inside	196258	ENSMUSG00000022306	Zfpm2
chr15	12031667	12032036	50,03	upstream	-15919	ENSMUSG00000022201	Zfr
chrY	1638548	1638782	132,56	upstream	-212191	ENSMUSG00000000103	Zfy2
chrY	1637354	1637864	190,29	upstream	-210997	ENSMUSG00000000103	Zfy2
chrY	1636895	1637289	138,28	upstream	-210538	ENSMUSG00000000103	Zfy2
chrY	1635740	1636029	67,39	upstream	-209383	ENSMUSG00000000103	Zfy2
chrY	1634884	1635342	467,60	upstream	-208527	ENSMUSG00000000103	Zfy2
chrY	1633554	1634008	187,37	upstream	-207197	ENSMUSG00000000103	Zfy2
chrY	1581658	1582043	80,91	upstream	-155301	ENSMUSG00000000103	Zfy2
chrY	1490331	1490503	314,37	upstream	-63974	ENSMUSG00000000103	Zfy2
chrY	1488516	1488728	90,13	upstream	-62159	ENSMUSG00000000103	Zfy2
chrY	1486956	1487584	878,23	upstream	-60599	ENSMUSG00000000103	Zfy2
chrY	1481358	1481873	355,73	upstream	-55001	ENSMUSG00000000103	Zfy2
chrY	1480994	1481329	95,23	upstream	-54637	ENSMUSG00000000103	Zfy2
chrY	1320773	1320920	98,82	downstream	105584	ENSMUSG00000000103	Zfy2
chrY	1314665	1314995	61,46	downstream	111692	ENSMUSG00000000103	Zfy2
chrY	1312707	1313056	94,88	downstream	113650	ENSMUSG00000000103	Zfy2
chrY	1311420	1311954	224,91	downstream	114937	ENSMUSG00000000103	Zfy2
chrY	1272136	1272304	199,57	downstream	154221	ENSMUSG00000000103	Zfy2
chrY	1267737	1268495	770,23	downstream	158620	ENSMUSG00000000103	Zfy2
chrY	1267356	1267702	135,71	downstream	159001	ENSMUSG00000000103	Zfy2
chrY	1266582	1266959	138,86	downstream	159775	ENSMUSG00000000103	Zfy2
chrY	1265965	1266418	138,24	downstream	160392	ENSMUSG00000000103	Zfy2
chrY	1265008	1265379	91,45	downstream	161349	ENSMUSG00000000103	Zfy2
chrY	1264190	1264474	666,90	downstream	162167	ENSMUSG00000000103	Zfy2
chrY	1263522	1263784	70,07	downstream	162835	ENSMUSG00000000103	Zfy2
chr6	92178734	92179113	57,42	upstream	-13815	ENSMUSG00000014550	Zfyve20
chr3	32230331	32230700	56,82	downstream	34051	ENSMUSG00000027663	Zmat3
chr8	114098204	114098637	56,57	inside	35961	ENSMUSG00000033545	Znrf1
chr16	55140610	55140894	58,42	downstream	142740	ENSMUSG00000064310	Zpld1
