# Supplementary material for: ChIP-Seq analysis identifies p27(Kip1)-target genes involved in cell adhesion and cell signalling in mouse embryonic fibroblasts
Source: PLoS One. 2017 Nov 20;12(11):e0187891. doi: 10.1371/journal.pone.0187891 (PMC5695801; doi:10.1371/journal.pone.0187891)
Supplement: S3 Fig — (PDF) [file pone.0187891.s003.pdf]

Cluster of Genes involved in Cell signaling

Ligands

Gene Name	Gene description
Bmp5	bone morphogenetic protein 5
Npvf	neuropeptide VF precursor
Nrg3	neuregulin 3
Hgf	hepatocyte growth factor
Wnt16	wingless-related MMTV integration site 16
Wnt2	wingless-related MMTV integration site 2
Gdnf	glial cell line derived neurotrophic factor
Il28a	interleukin 28A
Il7	interleukin 7
Edn1	endothelin 1
Efna5	ephrin A5
Fgf14	fibroblast growth factor 14
Fgf20	fibroblast growth factor 20
Lect1	leukocyte cell derived chemotaxin 1
Nxph1	neurexophilin+B68 1
Nxph2	neurexophilin 2
Vip	vasoactive intestinal polypeptide

Receptors

Gene Name	Gene description
Mrgprb3	MAS related GPR, member B3
hcrtr2 (OX-2)	hypocretin (orexin) receptor 2
Adrb2 (B2AR)	beta 2 adrenergic receptor
Sorl1	sortilin-related receptor, LDLR class A repeats-containing
FZD1	frizzled homolog 1 (Drosophila)
LPAR3	lysophosphatidic acid receptor 3
Ryr3	ryanodine receptor 3
Igf1r	insulin-like growth factor I B17 receptor
Msr1	macrophage scavenger receptor 1
Esrrg	estrogen-related receptor gamma
Npsr1	neuropeptide S receptor 1
Grid2	glutamate receptor, ionotropic, delta 2
Grik2	glutamate receptor, ionotropic, kainate 2 (beta2)
Grin2a	glutamate receptor, ionotropic, NMDA2A (epsilon 1)
Grin2b	glutamate receptor, ionotropic, NMDA2B (epsilon 2)
Grm1	Glutamate receptor metabotropic 1
Il12rb2	interleukin 12 receptor, beta 2
Il18rap	interleukin 18 receptor accessory protein
Il1rap	interleukin 1 receptor accessory protein
Epha3	Ephrin receptor A3
Epha5	Ephrin receptor A5
Epha6	Ephrin receptor A6
Rora	RAR-related orphan receptor alpha
Rorb	RAR-related orphan receptor beta
Adcyap1r1	adenylate cyclase activating polypeptide 1 receptor 1

G-proteins

Gene Name	Gene description
RhoT1	ras homolog gene family, member T1
Rhobtb1	Rho-related BTB domain containing 1
Rap2a	RAS related protein
Rerg	RAS-like, estrogen-regulated, growth-inhibitor
Arf4	ADP-ribosylation factor 4
Gnal	guanine nucleotide binding protein, alpha stimulating, olfactory type

Gnao	guanine nucleotide binding protein, alpha O
Gng4	guanine nucleotide binding protein (G protein), gamma 4

GEFs

Gene Name	Gene description
Akap13	A kinase (PRKA) anchor protein 13
Farp2	FERM, RhoGEF and pleckstrin domain protein 2
Rasgrp3	RAS, guanyl releasing protein 3
Rapgef5	Rap guanine nucleotide exchange factor (GEF) 5
Rapgef6	Rap guanine nucleotide exchange factor (GEF) 6

GAPs

Gene Name	Gene description
Rgs6	regulator of G-protein signaling 6
Rgs17	regulator of G-protein signaling 17
Arhgap15	Rho GTPase activating protein 15
Arhgap17	Rho GTPase activating protein 17
Ralgapa2	Ral GTPase activating protein, alpha subunit 2
Rasa1	RAS p21 protein activator 1
Syde2	synapse defective 1, Rho GTPase, homolog 2 (C. elegans)

Kinases

Gene Name	Gene description
MAPK10 (JNK3)	mitogen-activated protein kinase 10
MAP3K5 (ASK1)	mitogen-activated protein kinase kinase kinase 5
MAP3K8 (COT)	mitogen-activated protein kinase kinase kinase 8
YSK4	Yeast Sps1/Ste20-related kinase 4 (S. cerevisiae)
MAP4K4	mitogen-activated protein kinase kinase kinase kinase 4
STK3	serine/threonine kinase 3 (Ste20, yeast homolog)
Tab2	TGF-beta activated kinase 1/MAP3K7 binding protein 2
ADK	Adenosin kinase
Adrbk2	Adrenergic receptor kinase beta 2
Camk2a	calcium/calmodulin-dependent protein kinase II alpha
Camk2d	calcium/calmodulin-dependent protein kinase II delta
Kalrn	RhoGEF kinase, Kalirin
Cdk11b	cyclin-dependent kinase 11B
Cdk20	cyclin-dependent kinase 20
Cdk6	cyclin-dependent kinase 6
Nek10	NIMA (never in mitosis a)-related kinase 10
FER	fer (fms/fps related) protein kinase
Fyn	Fyn proto-oncogene
Hk1	hexokinase 1
Mark1	MAP/microtubule affinity-regulating kinase 1
Dgkh	diacylglycerol kinase, eta
Dck	deoxycytidine kinase
Prkch	protein kinase C eta
Prkd1	protein kinase D1
Pik3r1	PI3-kinase p85-alpha
Mylk	myosin, light polypeptide kinase
Mylk4	myosin light chain kinase 4
Smok2a	sperm motility kinase 2A
Strap	serine/threonine kinase receptor associated protein
Rps6kb1	ribosomal protein S6 kinase, polypeptide 1
Magi1	membrane associated guanylate kinase 1
Magi2	membrane associated guanylate kinase 2

Phosphatases

Gene Name	Gene description
Dusp26	dual specificity phosphatase 26
Inpp4b	inositol polyphosphate-4-phosphatase, type II
Ppfia2	protein tyrosine phosphatase, receptor type f
Ppm1a	protein phosphatase 1A, magnesium dependent, alpha isoform
Ppm1k	protein phosphatase 1K
Ppme1	protein phosphatase methylesterase 1
Ppp2r3a	protein phosphatase 2, regulatory subunit B,alpha
Ppp3ca	protein phosphatase 3, catalytic subunit, alpha
Ptprd	protein tyrosine phosphatase, receptor type D
Ptprg	protein tyrosine phosphatase, receptor type G
Ptprm	protein tyrosine phosphatase, receptor type M

Cyclases

Gene Name	Gene description
Guca2a	guanylate cyclase activator 2a (guanylin)
Gucy1a2	guanylate cyclase 1, soluble, alpha 2
