# Supplementary material for: ChIP-Seq analysis identifies p27(Kip1)-target genes involved in cell adhesion and cell signalling in mouse embryonic fibroblasts
Source: PLoS One. 2017 Nov 20;12(11):e0187891. doi: 10.1371/journal.pone.0187891 (PMC5695801; doi:10.1371/journal.pone.0187891)
Supplement: S1 Table — (PDF) [file pone.0187891.s004.pdf]

ChIP primers	Sequence (5´-3´)
Map3k5 fwd	GATGTGCATTCTACCCTGGAA
Map3k5 rev	CAGCAAGCATGAGAGCAAAA
Cxcl15 fwd	GCCACCAGGGAGGGTAGAAT
Cxcl15 rev	TTTCTGTGAGCCCATGCCAGT
Rasgrp3 fwd	GCTACATCCTCCCCTTCCTC
Rasgrp3 rev	CTTTAACCCCAGCACTCAGG
Hgf fwd	TATTTGTGCCCCTGGTTAGG
Hgf rev	GGCTTCTGGTCTGCACTTTC
Adamts9 fwd	CTCCTTACTGTGCCCTGAGC
Adamts9 rev	AAGGGAAGACCTGGAAAGGA
Pde7b fwd	CACTAAGGCGCACTGTGTGT
Pde7b rev	CTGGGAAGAGGGAATGTCAA

S1 Table
