# Supplementary material for: ChIP-Seq analysis identifies p27(Kip1)-target genes involved in cell adhesion and cell signalling in mouse embryonic fibroblasts
Source: PLoS One. 2017 Nov 20;12(11):e0187891. doi: 10.1371/journal.pone.0187891 (PMC5695801; doi:10.1371/journal.pone.0187891)
Supplement: S2 Table — (PDF) [file pone.0187891.s005.pdf]

Expression Primers	Sequence (5´-3´)
Gapdh fwd	ACCCAGAAGACTGTGGATGG
Gapdh rev	ACACATTGGGGTAGGAACA
Map3k5 fwd	GGCCGAATCTACAAAGATATG
Map3k5 rev	CTTTTTGAACCAAGATGCTC
Cxcl15 fwd	TTACTGCAACAGAAAGGAAG
Cxcl15 rev	GGTAGGAACCTGTTAGTAATTG
Rasgrp3 fwd	CCGAAGTTACAAAGAACTGG
Rasgrp3 rev	ATTTTGAAGCCATCACAGTC
Hgf fwd	CAAATGCAAGGACCTTAGAG
Hgf rev	CTTGTTTTGGATAAGTTGCC
Adamts9 fwd	GAGGATGACAACTACCTAGC
Adamts9 rev	GTACAGTTCAGTCTTTCCAC
Pde7b fwd	GAAATGACACAGGATATCGAAC
Pde7b rev	CTGAAGCATAAAGTGTCTGTC
Ctnnd2 fwd	ATGGCTCTGAGACGGAAACC
Ctnnd2 rev	CTGGCTACGATCTGGCGTTC
Col12a1 fwd	AGGCAGAAGTTGACCCACCT
Col12a1 rev	CAGTGGTACTAGCTGCAAGGG
Ncam1 fwd	AGAAATCAGCGTTGGAGAGTCC
Ncam1 rev	TCGTCATCATTCCACACCACT
Nedd9 fwd	TCAAGTGCCAAATTCCCAGG
Nedd9 rev	GTGCCGCCAATGTTCCTCT
Pcdh9 fwd	ACAGCCGCTTTCCAATCCC
Pcdh9 rev	TCCAAGTTCTGCTGAACAATCAA
Amigo2 fwd	CCCTGCCTAGAGCTGTCAAAC
Amigo2 rev	CAGTGGGGCACATTCCTGA
Itga8 fwd	TGTCTGGCGTTCAACTTGGAT
Itga8 rev	TCCAGTGAGTAGCCGAAGTAG

S2 Table
