# Supplementary material for: ChIP-Seq analysis identifies p27(Kip1)-target genes involved in cell adhesion and cell signalling in mouse embryonic fibroblasts
Source: PLoS One. 2017 Nov 20;12(11):e0187891. doi: 10.1371/journal.pone.0187891 (PMC5695801; doi:10.1371/journal.pone.0187891)
Supplement: S3 Table — (PDF) [file pone.0187891.s006.pdf]

Mef2c chr13:83037222-83037587>mm9

ACCTATCTCAAAATTTTTAACCCAGAAGTTTTACTGTCTAAAGGAAATG
CAGAGACAAAGAGTGGAGCAGAGACTGAGCAAAAGGCCACTCAGTGATC
ACCCTACCTCCTAGGTAGGTGCAGATCTATCCCACCTGCAGACACCAAA
CCCAGGCACTATTGCTGATGCCAAGAAGCACTTGGTGACAGGAGCCTCG
TATGGCTGATTACTCAGAGGTTCTACAAGCACCTATCCAATACAGATGC
ATATACTCACAGTCAACCATAGGACTGAACAAGGGGACCCCAATGGAAG
AGCGAGGAGAAGGACTGAAGGAGCTGAAGGAAATTGCAACCCTATAGGA
AGAACAATATCAACGAACTGGAC

Sox6 chr7:122987954-122988365>mm9

TGGTAACAGAAAACACAGAGAAGGATTATTTCAAAAACCTTCACGTAAGG
TGCAGCGTACATGCTCATATGGAGCCTCCAGCTACATCAAGCTGTAAGAA
GGTGAGGCCAGCCACGGTCGCCCATGTCAACTGCCTGCAGCAAGGCAGAG
GGATGAGTAAGACTACTGTGAGATGGCGCAATAGGTGACAGACTGTGCTG
GCAAGAGGTCACTGTACATGGGACAAAAAGCAAACCTCAGCCAGGGCCAG
TTGTTTGGCAGGAGGCCTTGCTTTTCAAAGTCCTGGCCTGACCCCTAAGC
ATGCCGCCCTGAACACCAGGTAGGTCCTCTGGGTCTCAGAGCCTTGACAT
GAAGCAGAAAACAACACATCAAATGGTTGAGCTTTTAAATTAATGCCCAA
ATTTGTATATAG

Shox2 chr3:66759083-66759484>mm9

AATGTTTGTGTTAGAGAAATAACTCTTTGTAAGGGGAACTAGTGCTATGG
TGTTTCCCAGAGGTTGATGTTTTGTGTGCCTGTTCCTCAGCTGATGGTAT
CGTTGGGGAAGGACCCTTTCAAAGACAGTGTTGGCTGGCAGCCACAGTCC
CTCCTGGGAAGCCAAAGCTAAGCCCCAGTGCCTGGCTCTCCTGCTGCACT
CTCTGCTCCTCCTTCTGTCCATGCTGAGAGCTGCAGCCACACATGCCCAT
CACCAGTGCTGAGTAGTACCTGCTCTGCCAGCATGAACTGAATCCCTCAG
AAATCATGAACCAAGATGGATTCTTCCCCTTTAAGCTGTCTGTTAGCTAT
TCTGTTACAAAAACAGCTAAGACAGAAATGGATGAAGAGTGTATTATACA
TT

Table S3
